# Analysis of the Mutational Landscape of Osteosarcomas Identifies Genes Related to Metastasis and Prognosis and Disrupted Biological Pathways of Immune Response and Bone Development

**DOI:** 10.3390/ijms241310463

**Published:** 2023-06-21

**Authors:** Sara Ferreira Pires, Juliana Sobral de Barros, Silvia Souza da Costa, Gabriel Bandeira do Carmo, Marília de Oliveira Scliar, André van Helvoort Lengert, Érica Boldrini, Sandra Regini Morini da Silva, Daniel Onofre Vidal, Mariana Maschietto, Ana Cristina Victorino Krepischi

**Affiliations:** 1 Human Genome and Stem-Cell Research Center, Institute of Biosciences, Department of Genetics and Evolutionary Biology, University of São Paulo, São Paulo 05508-090, Brazil; 2 Molecular Oncology Research Center (CPOM), Barretos Cancer Hospital, Barretos 14784-384, Brazil; 3 Barretos Children’s Cancer Hospital, Barretos 14784-400, Brazil; 4 Department of Pathology, Barretos Cancer Hospital, Barretos 14784-400, Brazil; 5 Brazilian Biosciences National Laboratory (LNBio), Brazilian Center for Research in Energy and Materials (CNPEM), Campinas 13083-884, Brazil

**Keywords:** osteosarcoma, chromothripsis, chromoanasynthesis, *RB1*, *TP53*, *PTPRQ*, *KNL1*, *ZFHX4*, *DMD*, *TNFRSF11B*

## Abstract

Osteosarcoma (OS) is the most prevalent type of bone tumor, but slow progress has been achieved in disentangling the full set of genomic events involved in its initiation and progression. We assessed by NGS the mutational spectrum of 28 primary OSs from Brazilian patients, and identified 445 potentially deleterious SNVs/indels and 1176 copy number alterations (CNAs). *TP53* was the most recurrently mutated gene, with an overall rate of ~60%, considering SNVs/indels and CNAs. The most frequent CNAs (~60%) were gains at 1q21.2q21.3, 6p21.1, and 8q13.3q24.22, and losses at 10q26 and 13q14.3q21.1. Seven cases presented CNA patterns reminiscent of complex events (chromothripsis and chromoanasynthesis). Putative *RB1* and *TP53* germline variants were found in five samples associated with metastasis at diagnosis along with complex genomic patterns of CNAs. *PTPRQ*, *KNL1*, *ZFHX4*, and *DMD* alterations were prevalent in metastatic or deceased patients, being potentially indicative of poor prognosis. *TNFRSF11B*, involved in skeletal system development and maintenance, emerged as a candidate for osteosarcomagenesis due to its biological function and a high frequency of copy number gains. A protein–protein network enrichment highlighted biological pathways involved in immunity and bone development. Our findings reinforced the high genomic OS instability and heterogeneity, and led to the identification of novel disrupted genes deserving further evaluation as biomarkers due to their association with poor outcomes.

## 1. Introduction

Osteosarcoma (OS) is the most prevalent malignant bone tumor in children and young people (0–19 years of age) [1,2], with an annual global incidence of 3.4 cases per a million individuals under the age of 15 years [3]. OS presents an aggressive clinical course with a high propensity for metastasis, especially in the lung [4,5]. Metastatic tumors are resistant to treatment, with a significant decrease in the survival rate [5,6]. These tumors most commonly develop near growth plates at the extremities of lower long bones such as the femur, but can also occur in other regions, including the jaw, humerus, pelvis, and tibia [5,7,8].

The annual incidence of OS is similar in the Brazilian and global populations according to previous studies, with respective rates of 4.13 cases (0–19 years) [9] and 4.4 cases per million (0–25 years) [5]. These tumors are slightly more common in males than females in Brazil as well in other populations, with a ratio of 1.24:1 in Brazil [10] and 1.43:1 globally [5]. The 5-year overall survival rates for patients with nonmetastatic disease are 59% in Brazil and 71.8% worldwide, but decrease substantially to 22% and 30.4%, respectively, for patients with metastasis detected at diagnosis [5,10]; these differences in overall survival rates can be attributed to poorer access to the public health system [11].

The main incidence peak of OS occurs concomitantly with the growth of the skeletal system during puberty, although a late onset form may occur in the elderly as a consequence of other cancers or Paget’s disease [2,5,12]. In Brazil, the median age at diagnosis is around 14 years old [10]. Since self-renewal, cell proliferation, and migration are common aspects of both organogenesis and tumorigenesis, disruptions affecting genes involved in cell cycle regulation, cell signaling, cell fate determination, hormonal regulation, etc., may contribute to the etiology of the disease [13,14].

The genomic complexity of OS makes it difficult to distinguish between driver and passenger alterations, impairing the development of molecular-tailored diagnosis and therapies [15,16]. Recurrent somatic mutations have been identified mainly in genes related to the maintenance of genomic stability, such as *ATRX*, *BRCA1*, *BRCA2*, *PTEN*, *RB1*, and *TP53* [6,15]. Germline pathogenic variants in genes such as *RB1*, *RECQL4*, and *TP53* (retinoblastoma, Rothmund–Thomson, and Li–Fraumeni syndromes, respectively) are known to increase the risk of OS development [6], accounting for approximately 28% of cases [17]. OSs are also characterized by complex karyotypes, evidenced by a high rate of amplifications, deletions, and translocations involving multiple regions or entire chromosomes [6,15]. The high genomic instability could be explained by complex events such as chromothripsis (chromosomal shattering followed by random rearrangement) and kataegis (regions of localized hypermutation), which have been observed in 30–90% and 50% of cases, respectively [5,6]. The epigenetic events related to tumor development and progression include global DNA hypomethylation and focal hypermethylation at CpG islands [18], which are events implicated in the activation of oncogenes or generation of genomic instability, and the silencing of tumor suppressors or other regulatory elements, respectively [19]. All these (epi)genomic changes detected in OSs might act synergistically to promote the disruption of key biological pathways related to the regulation of skeletal system morphogenesis, cell proliferation, cell fate, and maintenance of genomic stability [13,16,18,20].

In the present study, we explored different levels of genomic alterations from 28 Brazilian patients diagnosed with primary OS who had tumor samples collected before any systemic therapeutic interventions, with the aim to identify recurrently affected genes and biological processes. We disclosed the molecular profile of somatic and putative germline single nucleotide variants (SNVs), indels, and copy number alterations (CNAs), followed by protein–protein network enrichment based on the group of OS-altered genes. Alterations were also identified in previously reported OS-related genes, as well as in novel candidate OS genes.

## 2. Results

### 2.1. SNV and Indel Analysis

**Figure 1:**
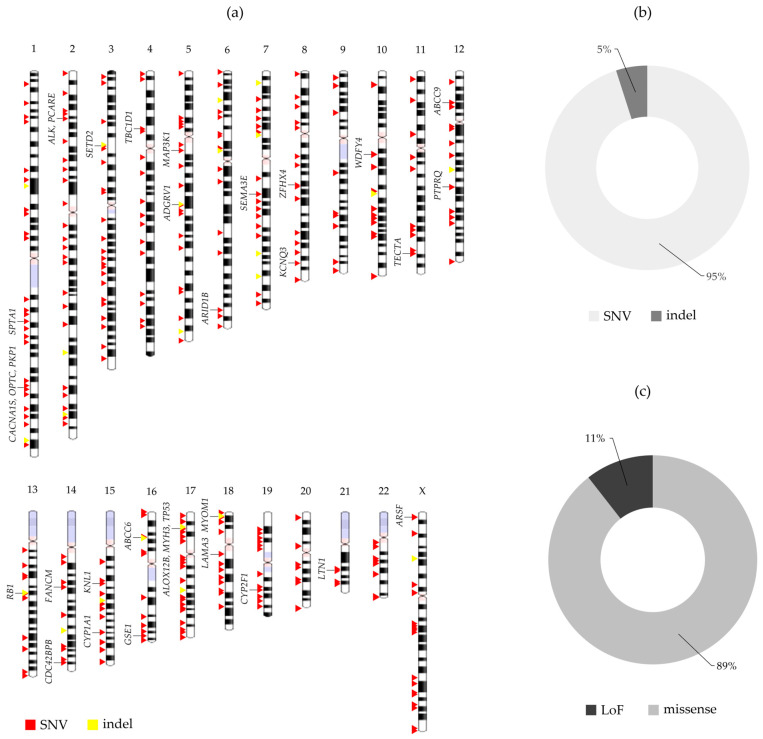
Characterization of the 445 coding non-synonymous somatic variants detected in the group of 28 primary osteosarcomas from Brazilian patients. (**a**) Genomic distribution of the identified SNV/indel variants across all chromosomes, indicated from 1 to 22 and X; red indicates SNVs and yellow, indels. The symbols of the recurrently mutated genes (with SNVs/indel variants detected in >1 patient) are depicted on the left side of the ideograms. (**b**) Characterization of the variants, according to their types: SNV—single nucleotide variant; indel—small insertion or deletion. (**c**) Characterization of the variants, according to their effects: missense—amino acid changes; LoF—loss-of-function.

A total of 74,880 SNVs and indels were detected in the 28 OS samples (average 2995 variants per tumor). After filtering for high-quality variants absent from populational variant databases, a total of 445 coding non-synonymous variants, which are putative somatic variants, were identified. These variants, mapped across nearly all chromosomes, are described below. Figure 1a illustrates the global profile of SNVs and indel variants identified in the group.

The identified variants were mapped to 400 genes (Supplementary Table S2a). Variants were identified in all samples, with an average of ~16 variants per tumor, ranging from 6 (OS-13) to 38 (OS-2) (median 14). The panel of variants was composed of 423 SNVs and 22 indels (Figure 1b), mostly resulting in amino acid changes (missense) (Figure 1c). Among them, 30 mutations were already reported in CIViC, 12 in COSMIC, 29 in ICGC, and 16 in TCGA; none of them were previously reported in OS samples according to these databases.

**Figure 2:**
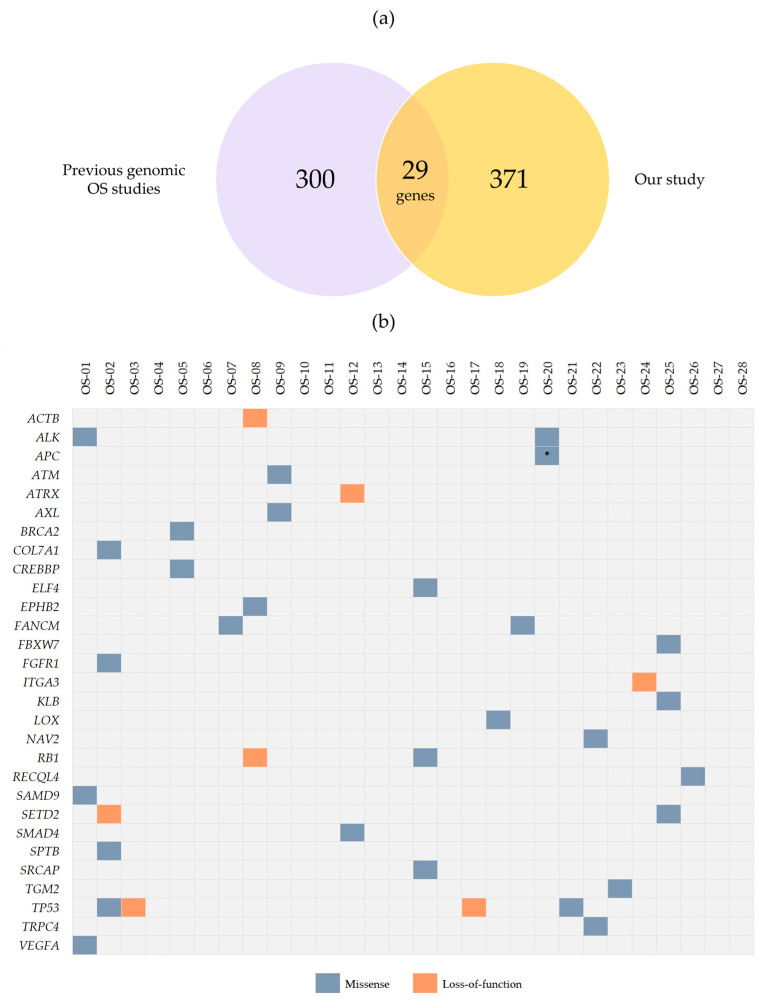
Overlapping genomic information between our study and previous genomic OS studies. (**a**) Venn diagram showing the genes common to the set of mutated genes in the Brazilian OS group and those reported in the literature. (**b**) Distribution of point mutations across 29 genes from our dataset and previously reported in the literature to be mutated in OS. The identified alterations are color-coded by type (missense or loss-of-function). Each row indicates a gene; each column indicates a tumor sample. The asterisk indicates the occurrence of more than one mutation in the same sample.

We compared these results with genes previously reported in the literature to be mutated in OS [5,21,22,23,24,25,26,27], deposited in the Cancer Gene Census from COSMIC [28], or known to be cancer predisposition genes [17,25,27,29,30,31]. Considering data from previous OS sequencing studies, 7.25% of overlapping genes were observed (29 genes, affected by 37 variants; Figure 2, Supplementary Table S2b): *ACTB*, *ALK*, *APC*, *ATM*, *ATRX*, *AXL*, *BRCA2*, *COL7A1*, *CREBBP*, *ELF4*, *EPHB2*, *FANCM*, *FBXW7*, *FGFR1*, *ITGA3*, *KLB*, *LOX*, *NAV2*, *RB1*, *RECQL4*, *SAMD9*, *SETD2*, *SMAD4*, *SPTB*, *SRCAP*, *TGM2*, *TP53*, *TRPC4*, and *VEGFA*.

We also annotated the complete list of 400 genes harboring SNVs/indels with both the Tumor Suppressor Genes database (https://bioinfo.uth.edu/TSGene/, accessed on 11 August 2022) and the Oncogene database (http://ongene.bioinfo-minzhao.org/, accessed on 11 August 2022), resulting in 43 tumor suppressors and 23 oncogenes (Table 1). We then compared the type of variant with the functional classification of the respective gene, searching for convergent information, emphasizing LoF variants for tumor suppressors and missense mutations for oncogenes. As a result, six tumor suppressors were observed with LoF mutations (*GNMT*, *MST1R*, *RB1*, *SETD2*, *TP53*, and *ZBTB16*). Nineteen missense mutations were mapped to oncogenes, five of which (*AXL*, *FGFR1*, *GLI2*, *TBC1D1*, and *TYRP1*) were classified as damaging according to at least five in silico algorithms. In particular, *TBC1D1* missense variants were detected in two OS samples.

Afterwards, 80 variants (mapped to 76 genes) were determined to have the highest likelihood of causing damage to the function of the protein. This included all LoF mutations and 33 missense mutations predicted to be deleterious by all applied in silico algorithms (Table 2). Of note, these mutations were observed almost exclusively in heterozygosity.

### 2.2. Recurrent Coding Non-Synonymous Variants

None of the SNV/indel variants were identified in more than one tumor. However, thirty-three genes were considered recurrently mutated (different variants detected in at least two OS samples; Figure 1a; Supplementary Table S2a): *ABCC6*, *ABCC9*, *ADGRV1*, *ALK*, *ALOX12B*, *ARID1B*, *ARSF*, *CACNA1S*, *CDC42BPB*, *CYP1A1*, *CYP2F1*, *FANCM*, *GSE1*, *KCNQ3*, *KNL1*, *LAMA3*, *LTN1*, *MAP3K1*, *MYH3*, *MYOM1*, *OPTC*, *PCARE*, *PKP1*, *PTPRQ*, *RB1*, *SEMA3E*, *SETD2*, *SPTA1*, *TBC1D1*, *TECTA*, *TP53*, *WDFY4*, and *ZFHX4*. *TP53* had the highest frequency of alterations (14.3% of the samples).

**Figure 3:**
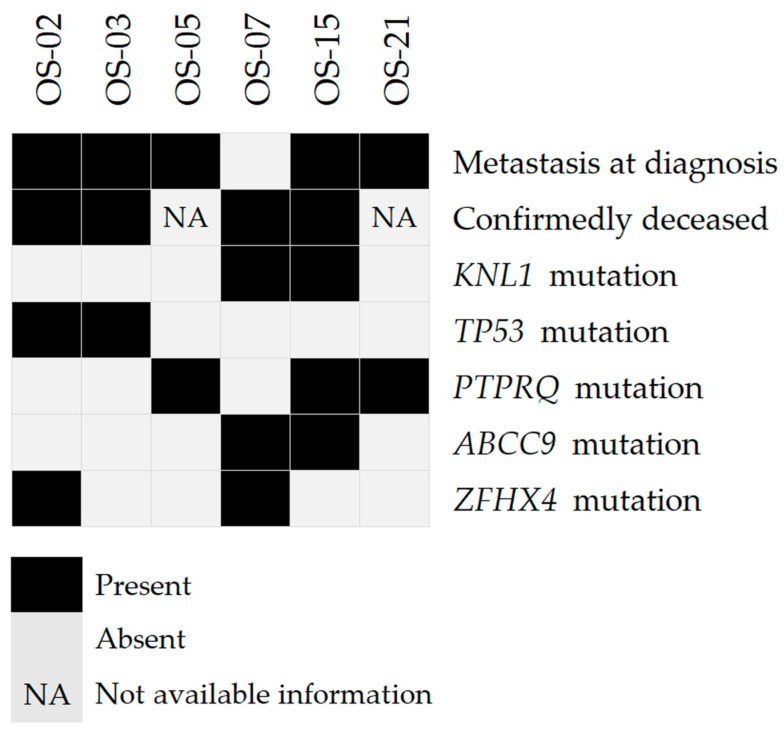
Distribution of point mutations across genes recurrently mutated in patients presenting clinical particularities.

Considering the clinical features of the OS group (Figure 3), we identified recurrently mutated genes in patients presenting metastasis at diagnosis (*TP53*—OS-2, OS-3, and OS-21; and *PTPRQ*—OS-5, OS-15, and OS-21) and patients who died from the disease (*TP53*—OS-2 and OS-3; *ABCC9* and *KNL1*—OS-7 and OS-15; and *ZFHX4*—OS-2 and OS-7). Only one patient without metastasis at diagnosis carried a *TP53* mutation (OS-17), while *PTPRQ* variants were detected only in metastatic patients. No gene was common in patients who were alive without the disease.

### 2.3. CNA

Our analysis identified 1176 CNAs in 27 of the 28 tumors, described in detail in Supplementary Table S3a. We detect 529 losses, 508 gains, 108 high copy gains (amplifications), and 31 homozygous losses, with an average number of 42 CNAs per sample, ranging from 0 (OS-18) to 90 (OS-4) per tumor (Supplementary Table S3b). The median size of the CNAs was 10.73 Mb (median 10.56 Mb for gains, 15.66 Mb for losses, 3.3 Mb for amplifications, 2.4 Mb for homozygous losses), ranging from 4 kb to 249.25 Mb (entire chromosomes).

**Figure 4:**
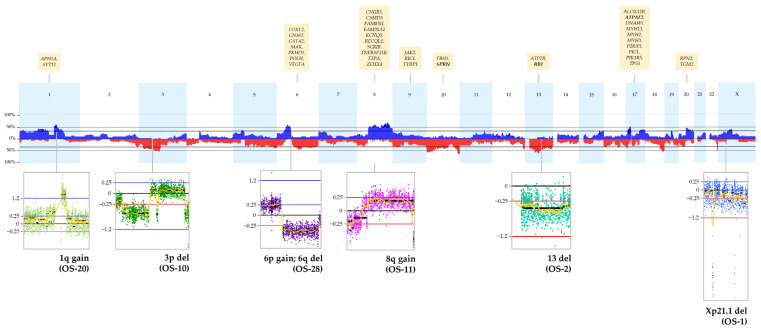
Global CNA profile of the 28 osteosarcomas cohort, with the respective frequencies of detected copy number gains and losses. On the x-axis, the chromosomes are indicated from 1 to 22 and X. The y-axis shows the detection frequency (%) of gains (in blue) and losses (in red) in the OS group. In the boxes below the graph, we highlighted specific CNAs, with the log_2_ sample/reference ratio thresholds indicated on the left of each figure: 0.25 for gains and 1.2 for high copy gains (blue lines), 0 for no differences in copy number (black line), −0.25 for losses and −1.2 for homozygous copy losses (red lines). In the same boxes, each dot represents one probe, and the colors of the dots are a way of representing different chromosomes. In the yellow boxes above the graph, we indicate the genes that, among those affected by SNV/indels, were the most recurrently affected by CNAs. The genes highlighted in **bold**, within these boxes, are the ones with the highest number of amplifications or homozygous copy losses. Images were obtained from Nexus Copy Number software version 9.0.

The OS samples displayed high CN heterogeneity, with multiple events spread over the entire genome. Figure 4 illustrates the global CNA profile identified in the group, highlighting alterations that are discussed below (the individual genomic profiles of the tumors are available in Supplementary Document S1). Aneuploidies involving entire chromosomes were mainly restricted to chromosome 13 (losses). Seven cases (OS-2, OS-4, OS-10, OS-11, OS-17, OS-23, and OS-25) presented losses of the whole chromosome 13 (in Figure 4, OS-2), while two (OS-8 and OS-19) lost nearly the entire chromosome; no common clinical features stood out among these patients. Considering chromosome arms, the most affected were 1q (partial gain), 3p (loss), 6p (gain), 6q (loss), and 8q (gain) (examples are shown in boxes of Figure 4).

All 400 genes with point mutations also harbored CNAs (Supplementary Table S3d). Of these, the genes with the highest number of alterations (≥15 alterations; Figure 3) were *FAM91A1*, *KCNQ3*, *TNFRSF11B* (20 alterations); *CSMD3*, *MYH13* (19 alterations); *DNAH9*, *FBH1*, *SPRN*, *ZFHX4* (18 alterations); *ALOX12B*, *APH1A*, *ATPAF2*, *GNMT*, *JAK2*, *MYH2*, *MYH3*, *PIK3R5*, *POLH*, *RIC1*, *TP53*, *TTPA*, *VEGFA* (17 alterations); *CNGB3*, *FAM83H*, *GSTA2*, *P2RX5*, *PIGL*, *PKHD1*, *RPN2*, *SCRIB*, *TYRP1* (16 alterations); and *ATP7B*, *FOXF2*, *MAK*, *RB1*, *RECQL4*, *SYT11*, *TGM2* (15 alterations).

**Figure 5:**
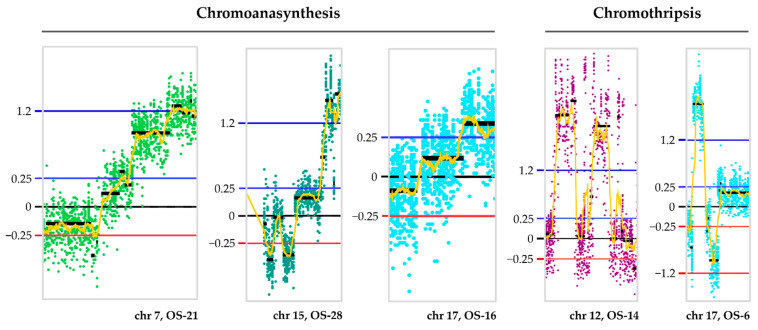
Complex chromosomal rearrangements revealed by CNA events identified in osteosarcomas. The type of event, chromoanasynthesis or chromothripsis, is indicated, as well as the affected chromosomes and sample IDs (x-axis), together with the log_2_ sample/reference ratio thresholds (y-axis): 0.25 for gains and 1.2 for high copy gains (blue lines), 0 for no differences in copy number (black line), −0.25 for losses and −1.2 for homozygous copy losses (red lines). Each dot represents one probe, and the colors of the dots are a way of representing different chromosomes. Images were obtained from Nexus Copy Number software version 9.0.

Five cases (OS-3, OS-15, OS-16, OS-21, and OS-28) presented a pattern of multiple localized amplifications of different copy number levels reminiscent of a complex rearrangement known as chromoanasynthesis. The affected chromosomes were 1 (OS-3), 7 (OS-21), 8, 14, 20 (OS-15), 15 (OS-28), and 17 (OS-16); three examples are given in Figure 5. All of them harbored alterations (mutations or losses) affecting DNA repair genes such as *TP53* (OS-3, OS-21, and OS-28), *RB1* (OS-15, OS-21, and OS-28), *BRCA2* (OS-15 and OS-21), and *PTEN* (OS-3).

Two cases (OS-14 and OS-19) stood out for presenting several contiguous alterations (gains and losses) grouped on a single chromosome (chromosome 12; example in Figure 5), with the rest of the genome involved in few CNAs. This localized complex fragmentation pattern is considered suggestive of the occurrence of chromothripsis. Similar fragmentation patterns were observed in the other two cases involving chromosomes 17 and 19 (OS-6 and OS-25; example in Figure 5), but the rest of the genome also presented multiple additional alterations (Supplementary Document S1). Almost all cases harbored copy number alterations affecting *TP53* (OS-6; loss), its regulator gene *MDM2* (OS-6, OS-19 and OS-25; gain/amplification), or DNA repair genes such as *BRCA1*/*BRCA2* (OS-19 and OS-25; loss), *PALB2* (OS-6 and OS-25; loss), *PTEN* (OS-6; loss) and *RB1* (OS-6, OS-19, and OS-25; loss).

Five cases (OS-1, OS-2, OS-6, OS-10, and OS-28) presented overlapping Xp21.1 focal losses (chrX:31685312-33957230, hg19; Figure 4 and Supplementary Figure S1), ranging from 880 kb to 2.27 Mb. This region encompasses a segment of *DMD*, including two noncoding genes, *MIR3915* and *MIR548F5*. These deletions were detected in samples from four males and one female, with ages from 13 to 20 years.

### 2.4. Recurrent CNA Events

Seventy-five CNAs presented a frequency ≥ 25% (*p*-value < 0.05; Supplementary Table S3c), encompassing 253 genes described in the Cancer Genes Census from COSMIC. The largest identified event was a common 1p36.33-p11.2 gain, with 121 Mb and encompassing 1542 genes; the smallest was a 4 kb gain at Xq13.3, encompassing only one gene, *MAGEE2*. The median length of the recurrent CNAs was 8.44 Mb (5.08 for gains, 17.34 for losses). In gains, 108 cancer genes were altered in at least 25% of the samples, including the oncogenes *CSF1*, *EVI5*, *ITGA3*, *JAK2*, *KIT*, *NCOA3*, *NTRK1*, *PPM1D*, and *TYRP1*. Eight genes presented CN gains and most likely pathogenic missense mutations (predicted as damaging by six in silico algorithms): *AQP3*, *CANT1*, *COL11A2*, *COL4A3*, *EPHB2*, *GH2*, *HOXA4*, and *SYT11*. A total of 118 genes were contained in deletions in at least 25% of the samples, including the tumor suppressors *APC*, *BAX*, *BRCA2*, *CDH13*, *CIC*, *CREBBP*, *CSMD1*, *IRF8*, *LOX*, *LZTS1*, *MST1R*, *PKD1*, *RB1*, *ROBO1*, *SERPINB5*, *SETD2*, *SMAD4*, and *TP53*. Twelve genes presented CN losses and LoF variants: *ABCC6*, *ADGRV1*, *ANO7*, *F12*, *MST1R*, *MYH11*, *PTGES2*, *RB1*, *RPS24*, *SCN10A*, *SETD2*, and *TP53*.

The most frequent CNAs, observed in more than 60% of our cohort, involved gains at 1q21.2q21.3 (160 genes), 6p21.1 (59 genes), and 8q13.3q24.22 (169 genes), and losses at 10q26.2q26.3 (62 genes) and 13q14.3q21.1 (32 genes) (Table 3 and Table S3e). The most recurrent event was the gain of 8q; although this region encompasses the *MYC* gene, the identified CNAs were very large chromosomal segments. Within the recurrent regions, we observed gains of the oncogenes *CDC5L*, *CUL7*, *DNPH1*, *MCL1*, *MLLT11*, *MYC*, *PCAT1*, *PTK7*, *PVT1*, *S100A4*, *S100A7*, *S100A8*, *SETDB1*, *TRIB1*, and *YWHAZ* and losses of the tumor suppressors *EBF3*, *MIR1297*, *OLFM4*, and *PCDH8.* However, none of them were affected by amplifications or homozygous losses.

The gene with the highest number of amplifications was *ATPAF2* (eight events), while the ones with the highest number of homozygous copy losses are *RB1* and *SPRN* (two events) (Figure 4). *ATPAF2* was also mapped to seven other gain events, while *RB1* and *SPRN* were deleted in 13 and 15 cases, respectively.

### 2.5. Candidate Germline Variants

A total of 34 SNVs and indel variants were mapped to 24 known cancer predisposition genes, all in heterozygosity (Supplementary Table S2c). A total of 5 of the 28 OSs (17.8%) harbored variants that met the criteria for germline evaluation according to the ESMO guidelines (Table 4); these variants were mapped to *RB1* (*n* = 2) and *TP53* (*n* = 3), with variant allele frequencies ranging from 43 to 81%.

The missense variant identified in *RB1* was previously classified in ClinVar as pathogenic/likely pathogenic (P/LP; variation ID 428682), identified in patients with hereditary retinoblastoma. Two of the three *TP53* LoF variants were already reported in other patients as pathogenic/likely pathogenic (ClinVar IDs 428908 and 182970), detected in both somatic and germline samples. Despite not being described in ClinVar, the frameshift variants of *RB1* and *TP53* were classified as LP following the ACMG guidelines.

Two of these patients, with mutations mapped to *TP53* (OS-3) and *RB1* (OS-15), carried chromosomal alterations reminiscent of chromoanasynthesis events and a CNA number higher than the cohort average (63 and 46, respectively). Moreover, considering the nine patients of the cohort who had metastasis identified at diagnosis, four of them (44.4%) carried a putative germline mutation.

### 2.6. Protein–Protein Interaction Network and Functional Enrichment Analysis

The resulting protein–protein interaction (PPI) networks from STRING (Supplementary Figure S2), generated from the list of proteins encoded by the OS-altered genes, revealed significantly more interactions between the groups than would be expected by chance, considering coding SNV/indel variants (*p*-value 2.23 × 10^−9^) or CNAs (*p*-value 6.99 × 10^−8^). The top 10 enriched biological processes (GO; Table 5) based on SNV/indels were related to development and homeostasis. In the KEGG analysis, the disrupted genes participate in similar signaling pathways, such as cell adhesion, ECM–receptor interaction, PI3K–Akt, immune cascades, pathways in cancer, hormone-related pathways, and regulation of calcium homeostasis. Among the input set of proteins, the ones presenting the highest number of connections, suggesting centrality among the disrupted group, were *CREBBP*, *ITGA2/3*, *PRKACA*, and *TP53*.

Since the identified CNAs consisted mostly of large events throughout the entire genome, we only considered the genes contained within regions of amplifications or deep deletions < 5 Mb (1677 genes) for the PPI analyses. The resulting network revealed enriched biological processes mostly related to the immune response, followed by developmental and metabolic processes (Table 5). Several enriched signaling pathways were consistently involved in immunity (e.g., JAK–STAT) and response to viruses such as HCV, HPV, MeV, EBV, and KSHV (Table 5). Our analysis also pointed to a direct interaction of several proteins with a neighboring member of the network (i.e., from outside the inputted list), *CCNA2.* Other proteins involved in multiple interactions (>10) were *TP53*, *AKT1*, *RB1*, *CDC42*, *CDK4*, *FOS*, *IRF3*, *HIF1A*, and *CCND2.* All of them, with the exception of *RB1*, were contained within amplification regions.

## 3. Discussion

Standard cancer therapeutic strategies, such as chemotherapy followed by surgical resection, have given OS patients higher survival rates [32]. However, most of them exert a nonspecific activity, e.g., DNA damage or cell growth inhibition, causing damage both to tumors and healthy cells since they directly disturb basic biological processes. Thus, approaches based on specific molecular targets are an interesting alternative for the development of less harmful, more accurate, and effective therapies.

OS is a complex and heterogeneous cancer type that presents a very particular genomic profile compared to other pediatric malignancies, including high mutational rates, genomic instability, and complex chromosomal rearrangements [8,13]. Overall, the group of tumors we analyzed displayed high genomic heterogeneity, as expected. Compared to other studies, our analyses identified a smaller average number of both SNVs/indels and CNAs [33,34], differences likely observed due to the platforms and algorithms used for the detection of alterations, together with the heterogeneity in the sample molecular composition. Only one sample had a small number of point mutations in combination with no identifiable CNAs, but this patient was not clinically different from the majority of the cases; we suppose this pattern is likely due to the heterogeneity intrinsic to this tumor type, as mentioned above. Grouping by age, we did not observe statistically significant differences in the mutational burden comparing pediatric/adolescent (up to 20 years old) to adults (over 20 years of age). However, this result can be ascribed either to the sample size (*n* = 28) or to differences in group sizes (23 pediatric/adolescent versus 5 adult patients).

We noted that only patients presenting metastasis at diagnosis had variants identified in protein tyrosine phosphatase receptor type Q (*PTPRQ)*. In the PedcBioPortal [35,36], ten OS patients carried mutations (mostly missense) affecting *PTPRQ*; four of them developed metastasis, and three of them died from the disease. In one case, different *PTPRQ* mutations were identified in both biopsy and relapse specimens from the same patient; in another case, two lung metastasis specimens from the same patient presented different *PTPRQ* mutations. *PTPRQ* encodes a protein from a family involved in important processes such as cell proliferation, differentiation, and survival [37,38]. Increased expression of *PTPRQ* was observed in metastatic tumors from mice [39]. Consistently, mutations resulting in the upregulation of other protein tyrosine phosphatase receptors have been implicated as a metastatic driver event in colorectal cancer [40] and cervical cancer [41,42]. Enhanced *PTPRQ* expression is described in the Human Protein Atlas (HPA) database (http://www.proteinatlas.org/, accessed on 11 May 2023) for several cancer groups, also including OS-derived cell lines. Taken together, these findings suggest that *PTPRQ* is an interesting predictive biomarker of poor prognosis for OS, although further functional validation is required to confirm this hypothesis.

Variants affecting kinetochore scaffold 1 (*KNL1)* were identified in two patients who died from the disease. The KNL1 protein is related to kinetochore–microtubule complex formation during chromosome segregation and interacts with other proteins that mediate the spindle assembly checkpoint during the cell cycle [43]. A total of 3 out of the 14 OS patients with samples reported in the PedcBioPortal [35,36] carrying *KNL1* mutations (mostly missense) are confirmedly deceased. In addition, one patient with OS (deceased) described in the St. Jude’s PeCan database [44] had the same somatic missense mutation affecting *KNL1* in different samples collected from metastatic tissue. Increased KNL1 protein expression has been reported in colorectal cancer and is significantly associated with poor survival, whereas the effects of its downregulation included inhibition of cell proliferation and induction of apoptosis [45]. High *KNL1* expression has been observed in all cancer cell lines available in the HPA database (accessed on 9 May 2023), of which five are from OS. However, there is still a lack of evidence for a role for *KNL1* in OS samples, highlighting this gene as a good candidate for further analysis.

Zinc finger homeobox 4 (*ZFHX4)* also had variants identified in two patients who died from cancer. In addition, the gene was highlighted among the ones with the highest number of CNAs (Figure 4), including gains or amplifications detected in 17 patients. From these, seven died from cancer (including the two who also had point mutations in *ZFHX4*). It has been suggested that mouse Zfhx4 participates in the transcriptional regulation of endochondral ossification during skeletal development, together with Osterix and Runx2; homozygous Zfhx4 knockout mice presented multiple skeletal deformities, and died as newborns [46]. *ZFHX4* has been proposed as a biomarker for poor prognosis in ovarian cancer [47,48,49] and esophageal squamous cell carcinoma [50]. *ZFHX4* mutations were also reported in 14 patients with OS described in the PedcBioPortal [35,36], with 4 of them being deceased. Although increased *ZFHX4* expression is documented in the HPA database (accessed on 10 May 2023) for several cancer types, it is higher in bone (including OS-derived cell lines) and rhabdoid cancers. Still, the role of *ZFHX4* in OS has not yet been investigated.

Somatic CNAs were observed to be substantially high in frequency and size, likely as a result of the increased genomic instability that is a hallmark of these tumors. Multiple CNAs have been reported in OS genomes, encompassing focal or large chromosomal segments, but it remains challenging to pinpoint specific genes with critical tumorigenic effects [15,16,26,34,51]. The most frequent alterations are gains at 1q, 6p, and 8q and losses at 10q and 13q, which are among the genomic events recurrently described in OSs [15,16,34,51]. Some of the genes encompassed by these CNAs are known cancer genes and have been previously associated with OS pathogenesis, such as *MYC*, *RB1*, and *RECQL4* [52,53].

We also identified copy number profiles consistent with complex chromosomal rearrangements in several cases, almost all of which contained some type of alteration in DNA repair or genomic stability maintenance genes, such as *BRCA2* and *TP53*. This finding indicates that these tumors may have a different initiation mechanism than others, most likely related to the generation of genomic instability and the dysregulation of basal cellular processes. The occurrence of a few catastrophic events may result in a more immediate, wide, and difficult-to-control impact than the gradual accumulation of small alterations in oncogenes or tumor suppressor genes [34,54].

Among the genes affected by CNAs, tumor necrosis factor receptor superfamily, member 11b (*TNFRSF11B)* stood out for presenting a high frequency of copy number increase (gains and high copy gains; 50 and 17.85%, respectively). The encoded protein osteoprotegerin (OPG) is involved in processes closely related to the development of the skeletal system [55]. Gene deletions or missense mutations resulting in a lack of protein activity have been implicated in the occurrence of juvenile Paget’s disease of the bone [56]. We hypothesize that excess *TNFRSF11B* copies may contribute to bone tumor development by increasing protein expression, leading to dysregulation of the balance between bone formation and resorption. OPG has been related not only to the regulation of bone morphogenesis but also to several processes involved in tumor development and progression, such as cell proliferation, epithelial to mesenchymal transition, angiogenesis, and invasion [57,58]. High *TNFRSF11B* expression has been recently described in the HPA database (accessed on 9 May 2023) for the OS cell line MG-63. Altogether, these pieces of evidence described above made us consider this gene a good candidate for OS development, as long as the sensitivity of the affected region to dose imbalance is evaluated [59,60].

Another intriguing recurrent CNA event was a focal loss of different sizes at Xp21.1, identified in four male patients and one female, always including an intragenic region of the *DMD* gene and the microRNAs *MIR3915* and *MIR548F5*. Four out of five patients carrying these Xp21.1 losses are deceased, and two had metastasis identified at diagnosis. Two patients (males, metastatic, deceased) reported in the PedcBioPortal [35,36] contained large Xp21.2-p21.1 losses encompassing the deletions identified here. Copy number losses or translocations encompassing the *DMD* gene have been described as a recurrent event in canine OS [61], and losses of *DMD* sequences have been suggested as a mechanism throughout human mesenchymal tumors evolve to more severe cases [62]. The role of the aforementioned microRNAs in cancer is not clear.

Moreover, potential germline variants in *RB1* and *TP53* were found in ~18% of the patients, some of them previously reported in ClinVar as germline alterations. As previously mentioned, two of the five (40%) patients harboring likely germline mutations presented complex chromosomal alterations and an increased CNA number; also, four of the nine (44.4%) metastatic patients carried one putative germline mutation. Thus, the presence of germline variants in cancer-predisposing genes could contribute to the tumor genomic instability and severity of these cases, and, conversely, the detection of specific clinical signs such as metastasis at diagnosis and complex genomic profiles could be biomarkers suggesting the presence of specific germline mutations. Given the possibility of immediate intervention in the clinical management of patients, our data reinforce the relevance of investigating the contribution of these mutations in patients with OS, even in the analysis of tumor-only sequencing data.

In addition to the relevance of potential germline variants in *TP53*, this gene was recurrently altered in our cohort, harboring SNV/indels in 14% and CNAs in 57% of the samples. Overall, *TP53* was disrupted in 60% of the group, a proportion that is consistent with worldwide studies conducted in OS samples. Considering only SNV/indels, these frequencies range in the literature from 17–26%, while including CNAs it increases to 35–82% [26,33,52]. Furthermore, ~18% of our patients presented variants maybe disrupting other known mechanisms of *TP53* inactivation, such as gains of *MDM2*. These alterations, either directly or indirectly affecting p53 activity, play a crucial role in osteosarcoma tumorigenesis, leading to genomic instability and the acquisition of other genetic abnormalities [13].

Despite the disclosed heterogeneity, the proteins in networks of interactions proved to be significantly connected (*p* < 0.05), with the main functional modules with enriched biological processes being related to development, response to stress and immunity. OSs arise in a rich microenvironment where highly specialized cells (including bone, vascular, stromal, and immune cells) mediate tissue formation and resorption in a dynamic and well-coordinated process. This environment is characterized by a high concentration of signaling components such as cytokines and growth factors, which is favorable for cancer development. In addition, tumor-associated macrophages (TAMs) are important and abundant immune components of the tumor microenvironment, and have a markedly recognized role in OS development, progression, maintenance, immunosuppression, and invasion [8,63,64]. Taken together, this information led to the use of immunomodulators such as mifamurtide, in combination with chemotherapy, in the treatment of OS, an approach that has been implied in the improvement of overall survival in OS patients [8,65].

It is noteworthy to mention that our analyses emphasized the significance of the PI3K–Akt pathway in both protein interaction networks (mutations and CNAs). This signaling pathway plays a critical role in various physiological processes (e.g., cell cycle regulation), and is frequently altered in OS [33,65,66]. Aberrant regulation of the PI3K/Akt pathway, especially hyperactivation, has been observed in both nonmetastatic and advanced-stage OSs [66]. Most importantly, PI3K inhibitors have emerged as promising agents for OS treatment, given their potential to suppress tumor progression and also increase the chemosensitivity of hard-responsive tumors to conventional therapies [33,67].

The networks also highlighted genes such as *CREBBP*, *ITGA2/3*, *PRKACA*, *TP53*, *AKT1*, *ITGAM*, *ITGB2*, *NTRK1*, *RB1*, *CDC42*, *CDK4*, *FOS*, *IRF3*, *HIF1A*, and *CCND2*, which act in processes including cell cycle regulation, metabolism, immune response, response to stress, differentiation, adhesion, and cell death. Several similar biological pathways have been previously found to be disrupted in OSs, such as pRb, p53, IGF, MAPK, MEK–ERK, mTOR, Notch, SHH, SMAD3, TGF-β, Wnt, and ALT [6,13,68,69,70]. Considering the biological context of disease onset, it is not surprising that alterations in cell growth, communication, differentiation, fate, and immune factors may contribute to the oncogenic development of OS [6,13,71].

A caveat of this study was the lack of paired normal and tumor samples, which was partially overcome by excluding from further analysis all variants reported in global and Brazilian populational databases, regardless of their frequency. We recognize that these databases do not harbor the complete genetic variability of human genomes, and it is possible that germline variants are present in the final set of putative “somatic” variants after the filtering process. Taking advantage of this limitation and also to address this concern, we conducted an additional analysis aiming to identify potential germline variants of clinical interest, according to the ESMO guidelines [72]. In addition, we did not have enough material available to carry out an extensive gene expression analysis that could confirm the impact of the aforementioned described genomic variants. We also considered applying computational pipelines to establish the signatures of DNA mutations and copy number alterations within our group; however, the platform we had available for performing tumor sequencing was not as robust as whole-genome or whole-exome sequencing to precisely infer aspects such as ploidy, loss of heterozygosity, and complex structural rearrangements.

Even so, our findings reinforced the high instability and heterogeneity of OS genomes and led to the identification of disrupted genes possibly linked to OS clinical features or development (*DMD*, *KNL1*, *PTPRQ*, *TNFRSF11B*, and *ZFHX4*), thus, contributing to the characterization of mechanisms likely involved in tumor development and progression that can be of interest for further investigation.

## 4. Materials and Methods

### 4.1. Characterization of the Patients

The cohort consisted of 28 patients (14 females, 14 males) diagnosed with primary OS. Fresh-frozen tumor samples collected before systemic treatment were recovered from the biobank of the Barretos Cancer Hospital (SP, Brazil). The clinical summary of these patients was previously published [18], and is available, with minor additions, as Supplementary Table S1. In short, 23 patients were diagnosed before the age of 20 (range of 10–20 years), and 5 were diagnosed after the age of 20 (29, 35, 37, 43, and 61 years); the median age at diagnosis was 17 years. Only one primary tumor was detected in each patient. Nine patients presented with metastasis at diagnosis; none of them had bone metastasis or skip metastasis detected during evaluation. Informed consent was obtained from the patients or their legal guardians, and the study was approved by the Ethical Research Committee of Barretos Cancer Hospital, Brazil, under the number CEP-HCB 898.403.

### 4.2. Clinical Exome Sequencing and Analysis 

The DNA libraries were prepared using a pre-enrichment strategy with the Nextera XT DNA Library Preparation kit (Illumina, Inc., San Diego, CA, USA, https://www.illumina.com/, accessed on 1 May 2023), followed by enrichment with the TruSight One panel (Illumina). This panel is focused on exonic regions (~62,000 target exons) and covers ~4800 disease-associated genes (12 Mb of genomic content). The enriched libraries were sequenced on the Illumina HiSeq 2000 platform, with a median depth coverage of 178x.

Raw reads were processed to remove adapters, low-quality bases (Phred score < 30), and reads < 35 bp using Sickle version 1.33 (https://github.com/najoshi/sickle), Scythe version 0.994 (https://github.com/vsbuffalo/scythe) and FastQC version 0.11.5 (https://www.bioinformatics.babraham.ac.uk/projects/fastqc/). After quality control inspection, the remaining reads were mapped to the reference genome version GRCh37 hg19 (http://hgdownload.cse.ucsc.edu/downloads.html) using Burrows–Wheeler Aligner (BWA) version 0.7.8 (https://github.com/lh3/bwa) [73]. The alignment data were converted to .bam files using SAMtools version 1.9 (https://github.com/samtools/samtools) [74]; the tools MarkDuplicates and CalculateHsMetrics from Picard version 1.81 (https://github.com/broadinstitute/picard) were used to remove PCR duplicates and to evaluate data distribution and read coverage. Local realignment of indels and recalibration of bases were performed using the tools RealignerTargetCreator and IndelRealigner from GATK version 3.7 (https://gatk.broadinstitute.org/) [75]. Variant calling was performed using the UnifiedGenotyper tool from GATK, resulting in .vcf files used for annotation and filtering. Access dates were on 1 May 2023.

The mutational burden was analyzed using the software VarSeq™ version 2.2.1 (Golden Helix, Inc., Bozeman, MT, USA, available from www.goldenhelix.com). The parameters applied to filter high-quality SNVs and indels were minimum base coverage of 20× and Phred quality score ≥ 17. For variant annotation, we used (a) the reference sequence database RefSeq Genes version 3.1 (https://www.ncbi.nlm.nih.gov/refseq/) [76]; (b) population databases of genomes and exomes such as 1000 Genomes (https://www.internationalgenome.org/) [77], gnomAD version 2.1.1 (https://gnomad.broadinstitute.org/) [78], ABraOM version SABE609 (https://abraom.ib.usp.br/) [79], dbSNP 154 version 2 (https://www.ncbi.nlm.nih.gov/snp/) [80], UK10K (https://www.uk10k.org/) [81], and NHLBI version 0.0.30 (https://www.nhlbi.nih.gov); (c) the cancer databases COSMIC (https://cancer.sanger.ac.uk/cosmic) [28], CIViC (https://civicdb.org/) [82], ICGC (https://dcc.icgc.org/) [83], and TCGA (https://www.cancer.gov/ccg/research/genome-sequencing/tcga) [84], and (d) the clinical databases ClinVar (https://www.ncbi.nlm.nih.gov/clinvar/) [85], and OMIM (https://www.omim.org/). Access dates were on 1 May 2023.

Coding somatic variants were filtered based on variant allele frequency ≥10%, absence in the population databases mentioned above, and effect (missense or loss-of-function (LoF)—frameshift, stop gain/loss, splicing mutation). Variants reported in the population databases were excluded to overcome the absence of matched germline samples. Variants mapped to hypervariable regions of the exome were also excluded [86]. All remaining filtered variants were verified by visual inspection of the .bam files. Pathogenicity predictions were obtained for missense variants from dbNSFP Functional Predictions version 3.0 (http://sites.google.com/site/jpopgen/dbNSFP) [87], which contains six in silico algorithms (SIFT, Polyphen2 HVAR, MutationTaster, MutationAssessor, FATHMM, and FATHMM MKL Coding). Access dates were on 1 May 2023.

For the detection of pathogenic variants of potential germline origin, we followed the guidelines of the European Society of Medical Oncology (ESMO) [72]. The variants were filtered by (1) allele frequency >20% for indels and >30% for SNVs, (2) pathogenicity (pathogenic or likely pathogenic based on ClinVar classification, or LoF), and (3) occurrence in one of the 27 high-risk and ‘actionable’ cancer predisposition genes [72].

### 4.3. CNA Analysis

CNA analysis was performed using the software Nexus Copy Number Discovery version 9.0 (Bionano Genomics, Inc., San Diego, CA, USA, available from https://bionano.com/nexus-copy-number-software/, accessed on 27 April 2022). The genome reference was built from .bam files of control samples that underwent sequencing on the same platform (TruSight One panel). This reference was then used as a baseline for CNA calling using the FASST2 segmentation algorithm (hidden Markov statistical model). CNA calls were based on a sample/reference threshold (measured as a log_2_ function) of ≥0.25 for gains, ≤−0.25 for losses, ≥1.2 for high copy gains, and ≤−1.2 for homozygous copy losses, affecting ≥15 consecutive probes. CNAs completely overlapping and covered by common CNVs on the Database of Genomic Variants [88] were disregarded. All automatically called variants were manually inspected for validation. The frequency of specific CNAs in the entire cohort was evaluated to determine recurrent alterations with statistical significance.

### 4.4. Protein–Protein Interaction (PPI) Network and Functional Enrichment Analysis of OS-Altered Genes

The STRING tool (version 11.5, https://string-db.org/, accessed on 2 February 2023) [89] was applied to establish a protein–protein interaction (PPI) network using the most recurrent OS-altered genes and close neighbors as seeds and the whole genome as background. All evidence types of interaction were considered, with a minimum confidence level of 0.9 and a maximum number of five direct interactors from outside the query list. The number of connections for each protein of interest was used to infer the relevance of the genes within the network. Enrichments for biological processes (Gene Ontology) and pathways (KEGG) were obtained, and features with false discovery rate (FDR) ≤ 0.05 were considered significant; the resulting terms were ranked in ascending order based on FDR values.

## Figures and Tables

**Table 1:** List of tumor suppressors and oncogenes containing coding SNVs and small indels.

Classification *	Genes
Tumor suppressors	*ADAMTS18*, *APC*, *ATM*, *AXIN2*, *BAX*, *BRCA2*, *CDH13*, *CIC*, *CREBBP*, *CSMD1*, *DCDC2*, *DNMT1*, *EPHB2*, *FBXW7*, ***GNMT***, *HRG*, *IRF8*, *ITGA7*, *LIFR*, *LOX*, *LZTS1*, *MAX*, ***MST1R***, *MYO18B*, *PAWR*, *PIN1*, *PKD1*, *PPP2R1B*, *PRDM5*, *PTPN13*, ***RB1***, *ROBO1*, *SCRIB*, *SERPINB5*, ***SETD2***, *SMAD4*, *SYNM*, *TGFBR3*, *THBS1*, ***TP53***, *VEGFA*, ***ZBTB16***, *ZFHX3*
Oncogenes	***ALK***, ***AXL***, ***BAX***, ***BCL11A***, ***CSF1***, ***ELF4***, ***EVI5***, ***FGFR1***, ***GLI2***, *ITGA3*, ***JAK2***, ***KIT***, *MBD1*, ***MEIS1***, *MST1R*, ***NCOA3***, ***NTRK1***, ***PPM1D***, ***RALGDS***, ***RHO***, ***TBC1D1***, ***TYRP1***, *ZBTB16*

*: Reference lists of human tumor suppressors and oncogenes were obtained from the Tumor Suppressor Genes database (https://bioinfo.uth.edu/TSGene/, accessed on 11 August 2022) and the Oncogene database (http://ongene.bioinfo-minzhao.org/, accessed on 11 August 2022). The genes highlighted in **bold** presented mutations consistent with their functional roles (LoF mutations in tumor suppressors, missense mutations in oncogenes).

**Table 2:** Description of most likely pathogenic SNV/indel mutations in the 28 tumors (LoF variants and missense variants predicted as damaging by 6/6 in silico tools).

Variant Info ^#^			RefSeq Genes 105 Interim v3.1, NCBI		OS Genes ^b^	Cancer Databases			
Genomic Coordinates (Chr:Start-Stop) ^a^	Ref/Alt Alleles	VAF	Gene Name	Effect	Match	COSMIC	ICGC	CIViC	TCGA
chr1:197316605-197316605	G/A	0.56	*CRB1*	LoF					
chr1:76215170-76215174	AAAGA/-	0.52	*ACADM*	LoF					
chr2:169761127-169761127	G/A	0.33	*G6PC2*	LoF					
chr2:188331670-188331669	-/T	0.75	*TFPI*	LoF					
chr2:227661111-227661111	C/A	0.15	*IRS1*	LoF					
chr2:227896863-227896871	CCTGGGGGT/-	0.53	*COL4A4*	LoF					
chr2:234201046-234201046	A/T	0.16	*ATG16L1*	LoF					
chr2:242149051-242149051	G/T	0.55	*ANO7*	LoF					
chr3:38798294-38798294	G/T	0.26	*SCN10A*	LoF					
chr3:47061249-47061249	C/A	0.52	*SETD2* ^TS^	LoF	Y				
chr3:49929189-49929189	A/G	0.38	*MST1R* ^TS/OG^	LoF					
chr4:100350727-100350727	C/A	0.5	*ADH7*	LoF					
chr5:176830344-176830343	-/T	0.38	*F12*	LoF					
chr5:89979528-89979527	-/T	0.46	*ADGRV1*	LoF					
chr6:42931340-42931340	G/T	0.75	*CNPY3-GNMT*, *GNMT*	LoF					
chr6:52617789-52617789	C/-	0.48	*GSTA2*	LoF					
chr7:107198514-107198514	A/C	0.6	*COG5*	LoF					
chr7:121726081-121726081	C/-	0.5	*AASS*	LoF					
chr7:138406691-138406691	T/-	0.73	*ATP6V0A4*	LoF					
chr7:40174717-40174717	C/G	0.45	*SUGCT*	LoF					
chr7:44561787-44561787	C/-	0.67	*NPC1L1*	LoF					
chr7:5569193-5569193	G/-	0.45	*ACTB*	LoF	Y				
chr8:77761365-77761365	G/T	0.65	*ZFHX4*	LoF					
chr9:130885414-130885414	C/A	0.22	*PTGES2*	LoF					
chr10:79795134-79795135	AG/-	0.32	*RPS24*	LoF					
chr10:79795137-79795137	A/T	0.31	*RPS24*	LoF					
chr11:114057673-114057673	G/A	0.93 (*)	*ZBTB16* ^TS/OG^	LoF					
chr11:93545017-93545017	A/C	0.37	*MED17*	LoF					
chr12:40645036-40645036	G/T	0.4	*LRRK2*	LoF					
chr12:66788074-66788073	-/C	0.51	*GRIP1*	LoF			Y		
chr13:48955486-48955486	T/-	0.45	*RB1* ^TS^	LoF	Y			Y	
chr15:50904973-50904972	-/TA	0.26	*TRPM7*	LoF					
chr15:53889390-53889390	G/A	0.18	*WDR72*	LoF					
chr15:84566757-84566757	G/T	0.31	*ADAMTSL3*	LoF					
chr16:15850335-15850335	C/A	0.22	*MYH11*	LoF			Y		Y
chr16:16315529-16315528	-/A	0.28	*ABCC6*	LoF					
chr17:48149353-48149353	G/A	0.43	*ITGA3* ^OG^	LoF	Y				
chr17:48268206-48268206	G/-	0.36	*COL1A1*	LoF					
chr17:7574003-7574003	G/A	0.7	*TP53* ^TS^	LoF	Y	Y	Y	Y	Y
chr17:7578190-7578189	-/T	0.81	*TP53* ^TS^	LoF	Y			Y	
chr17:7578370-7578370	C/T	0.43	*TP53* ^TS^	LoF	Y	Y	Y	Y	Y
chr18:3193953-3193959	AAGTCTG/-	0.57	*MYOM1*	LoF					
chr18:39637927-39637927	G/T	0.18	*PIK3C3*	LoF					
chr18:47806245-47806245	C/T	0.47	*MBD1* ^OG^	LoF					
chrX:31089722-31089722	C/-	0.96 (**)	*FTHL17*	LoF					
chrX:57318934-57318934	A/T	0.34	*FAAH2*	LoF					
chrX:76939760-76939760	T/A	0.28	*ATRX*	LoF	Y			Y	
chr1:155851183-155851183	G/T	0.23	*SYT11*	Missense			Y		
chr1:225594417-225594417	G/A	0.55	*LBR*	Missense					
chr1:23234503-23234503	G/C	0.14	*EPHB2* ^TS^	Missense	Y			Y	
chr1:237802396-237802396	G/C	0.2	*RYR2*	Missense					
chr2:116447461-116447461	G/T	0.22	*DPP10*	Missense			Y		
chr2:169781238-169781238	C/T	0.55	*ABCB11*	Missense			Y		
chr2:228154801-228154801	C/A	0.2	*COL4A3*	Missense					
chr2:234343485-234343485	C/G	0.44	*DGKD*	Missense					
chr2:26501668-26501668	A/G	0.48	*HADHB*	Missense					
chr3:164764634-164764634	G/T	0.17	*SI*	Missense					
chr3:4558263-4558263	T/G	0.35	*ITPR1*	Missense					
chr5:78181435-78181435	C/T	0.58	*ARSB*	Missense					
chr6:157502299-157502299	G/A	0.12	*ARID1B*	Missense					
chr6:161139813-161139813	T/A	0.23	*PLG*	Missense					
chr6:33143809-33143809	C/A	0.17	*COL11A2*	Missense					
chr7:27169095-27169095	G/C	0.57	*HOXA4*	Missense					
chr8:38272404-38272404	G/T	0.25	*FGFR1* ^OG^	Missense	Y		Y	Y	
chr9:33447464-33447464	A/G	0.41	*AQP3*	Missense					
chr10:105792709-105792709	C/T	0.85	*COL17A1*	Missense			Y		
chr11:119213431-119213431	C/A	0.54	*C1QTNF5*, *MFRP*	Missense					
chr12:21964993-21964993	C/A	0.23	*ABCC9*	Missense					
chr13:113793695-113793695	C/T	0.22	*F10*	Missense					
chr13:52544680-52544680	C/T	0.33	*ATP7B*	Missense		Y	Y		Y
chr14:103371559-103371559	A/C	0.57	*TRAF3*	Missense					
chr14:103418914-103418914	C/A	0.31	*CDC42BPB*	Missense					
chr16:3781285-3781285	C/T	0.27	*CREBBP* ^TS^	Missense	Y			Y	
chr17:61958178-61958178	T/C	0.52	*GH2*	Missense					
chr17:7577124-7577124	C/G	0.74	*TP53* ^TS^	Missense	Y	Y	Y	Y	
chr17:76989711-76989711	G/C	0.43	*CANT1*	Missense					
chr19:14208479-14208479	T/C	0.31	*PRKACA*	Missense				Y	
chr19:33716477-33716477	C/A	0.62	*SLC7A10*	Missense					
chr19:41759546-41759546	G/A	0.18	*AXL* ^OG^	Missense	Y		Y	Y	Y
chrX:138623259-138623259	C/T	0.45	*F9*	Missense			Y		

^#^: None of the described variants were identified in more than one tumor; ^a^: reference genome GRCh37 hg19; ^b^: custom gene list for inspection of genes previously reported in osteosarcomas sequencing studies; (*): alterations in homozygosis for non-reference alleles; (**): alterations in hemizygosis for non-reference alleles; ^TS^: tumor suppressor gene, according to the Tumor Suppressor Genes database; ^OG^: oncogene, according to the Oncogene database. Pathogenicity predictions were obtained from dbNSFP functional predictions; the score indicates the number of in silico algorithms that predicted the variant as pathogenic (SIFT, Polyphen2 HVAR, MutationTaster, MutationAssessor, FATHMM, and FATHMM MKL Coding). Abbreviations: VAF—variant allele frequency; LoF—loss-of-function.

**Table 3:** Description of the five most recurrent CNAs detected in ≥60% of the cohort of 28 osteosarcomas.

Region *	Length (Mb)	Cytoband Location	Event	Genes	miRNAs	Cancer Gene Census ^#^
chr1:149683910-153535697	3.85	q21.2–q21.3	Gain	160	2	*ARNT*
chr6:42848233-45479933	2.64	p21.1	Gain	59	1	
chr8:71415111-133492411	28.43	q13.3–q24.22	Gain	169	12	*EXT1*, *MYC*
chr10:128038823-135454121	7.41	q26.2–q26.3	Loss	62	3	
chr13:53415739-57786339	4.37	q14.3–q21.1	Loss	32	1	

*: Genomic coordinates are indicated according to the reference genome GRCh37 hg19; #: Cancer Gene Census is a section from Catalogue of Somatic Mutations in Cancer (COSMIC) composed by genes containing driver mutations in cancer.

**Table 4:** Variants that meet the criteria for germline evaluation, according to the ESMO guidelines.

Gene	Variant (GRCh37/hg19)	ID	Read Depth	VAF (%)	ClinVar ID	ClinVar Classification	Franklin Classification	ACMG Criteria *
*RB1*	chr13:48937089-A/G	OS-15	53	55	428682	P/LP	LP	PM2, PP3, PP5
	chr13:48955486:T/-	OS-8	63	45	-	-	LP	PVS1, PM2
*TP53*	chr17:7578370-C/T	OS-3	23	43	428908	P/LP	P	PVS1, PM2, PP5
	chr17:7574003-G/A	OS-17	40	70	182970	P	P	PVS1, PM2, PM1, PP5
	chr17:7578190:-/T	OS-2	32	81	-	-	LP	PVS1, PM2

*: Aggregated evidence from Franklin ACMG Classification Tool. Abbreviations: VAF—variant allele frequency; LP—likely pathogenic; P—pathogenic.

**Table 5:** Most significantly enriched GO biological processes and KEGG signaling pathways of the OS-altered genes, sorted by false discovery rate (↑).

Type of Alteration	GO	KEGG
**Coding SNVs and indels** 400 inputted genes 405 nodes 218 edges	Multicellular organismal process Anatomical structure development Developmental process Multicellular organism development Regulation of biological quality System development Cellular component organization Homeostatic process Anatomical structure morphogenesis Animal organ development	Pathways in cancer PI3K–Akt signaling pathway ECM–receptor interaction Human papillomavirus infection Small cell lung cancer Arrhythmogenic right ventricular cardiomyopathy Focal adhesion Proteoglycans in cancer Dilated cardiomyopathy Hypertrophic cardiomyopathy
**Copy number alterations** 1677 inputted genes 1132 nodes 582 edges	Positive regulation of peptidyl-serine phosphorylation of stat protein Regulation of peptidyl-serine phosphorylation of stat protein Natural killer cell activation involved in immune response Response to exogenous dsiRNA B-cell proliferation Humoral immune response T-cell activation involved in immune response Lymphocyte proliferation Leukocyte proliferation Negative regulation of glucuronosyltransferase activity	Autoimmune thyroid disease Cytosolic DNA-sensing pathway RIG-I-like receptor signaling pathway JAK–STAT signaling pathway Hepatitis B Epstein–Barr virus infection Ascorbate and aldarate metabolism Toll-like receptor signaling pathway Human papillomavirus infection PI3K–Akt signaling pathway

Abbreviations: GO—Gene Ontology; KEGG—Kyoto Encyclopedia of Genes and Genomes; FDR—false discovery rate.

## Data Availability

The data presented in this study are available on request from the corresponding author.

## Supplementary Materials

The following supporting information can be downloaded at https://www.mdpi.com/article/10.3390/ijms241310463/s1.
