# Supplementary material for: Analysis of the Mutational Landscape of Osteosarcomas Identifies Genes Related to Metastasis and Prognosis and Disrupted Biological Pathways of Immune Response and Bone Development
Source: Int J Mol Sci. 2023 Jun 21;24(13):10463. doi: 10.3390/ijms241310463 (PMC10342084; doi:10.3390/ijms241310463)
Supplement: Supplementary file 1 [file ijms-24-10463-s001.zip › Supplementary Table S1.pdf]

Supplementary Table S1. Clinical data of the 28 osteosarcoma patients and mutational summary.

ID	Age	Sex	Histology	Metastasis	Death status (cause)	Huvos grade	Tumor size (cm)	SNV/ indel	CNA
OS-1	14	M	Mixed	-	Deceased (other)	1	15	29	63
OS-2	13	M	Chondroblastic	+	Deceased (cancer)	1	13	38	46
OS-3	18	M	Chondroblastic	+	Deceased (cancer)	1	NA	16	63
OS-4	37	F	Osteoblastic	NA	NA	NA	NA	22	90
OS-5	17	M	Osteoblastic	+	NA	1	18.8	13	59
OS-6	17	M	Telangiectatic	NA	Alive without cancer	NA	10.7	11	75
OS-7	20	M	Osteoblastic	-	Deceased (relapse)	2	NA	25	23
OS-8	16	F	Telangiectatic	+	Deceased (other)	1	9.4	11	32
OS-9	18	F	Osteoblastic	+	Deceased (cancer)	2	9.9	14	18
OS-10	19	F	Osteoblastic	+	Deceased (cancer)	2	5	17	64
OS-11	12	M	Osteoblastic	-	Alive without cancer	1	18.3	16	28
OS-12	29	F	Osteoblastic	-	NA	1	28	30	27
OS-13	13	M	NA	+	Alive without cancer	NA	18.3	6	38
OS-14	19	F	Parosteal	-	NA	NA	15.4	9	11
OS-15	11	F	Osteoblastic	+	Deceased (cancer)	1	10.5	18	46
OS-16	43	M	Osteoblastic	-	NA	2	NA	11	19
OS-17	18	F	Osteoblastic	-	Alive without cancer	2	22	19	14
OS-18	12	F	Osteoblastic	+	Deceased (cancer)	2	12	13	0
OS-19	17	F	Fibroblastic	-	Alive without cancer	1	NA	10	18
OS-20	17	M	Osteoblastic	+	Deceased (cancer)	1	14.2	18	25
OS-21	35	M	Osteoblastic	+	NA	1	NA	14	52
OS-22	18	F	Osteoblastic	-	Deceased (other)	2	33	15	17
OS-23	13	F	Osteoblastic	-	Deceased (cancer)	1	10	10	66
OS-24	15	F	Osteoblastic	-	Deceased (other)	1	17.5	15	65
OS-25	61	M	Pleomorphic	-	NA	NA	NA	8	54
OS-26	17	M	Osteoblastic	-	Deceased (cancer)	2	9.1	13	42
OS-27	10	F	Osteoblastic	-	Deceased (other)	1	18	14	48
OS-28	20	M	Osteoblastic	-	Deceased (cancer)	1	16	10	73

(Abbreviations) F: female; M: male; NA: not available; SNV: single nucleotide variant; CNA: copy number alteration.
