# Supplementary material for: Analysis of the Mutational Landscape of Osteosarcomas Identifies Genes Related to Metastasis and Prognosis and Disrupted Biological Pathways of Immune Response and Bone Development
Source: Int J Mol Sci. 2023 Jun 21;24(13):10463. doi: 10.3390/ijms241310463 (PMC10342084; doi:10.3390/ijms241310463)
Supplement: Supplementary file 1 [file ijms-24-10463-s001.zip › List of Supplementary Material.pdf]

List of Supplementary Material

Supplementary Figure S1. Five cases of focal Xp21.1 losses. Samples are ordered by chromosomal starting position and each red bar indicates one sample. A 683 kb overlapping common region among the deletions was delimited (chrX:32311411-32994615, highlighted in light blue). Data were generated and analyzed using the UCSC Genome Browser on Human Feb. 2009 (GRCh37/hg19) Assembly.

Supplementary Figure S2. Protein–protein interaction (PPI) networks using the proteins encoded by genes containing **(a)** SNVs and small indels, or **(b)** copy number alterations, with minimum confidence level of 0.9. Networks were generated using STRING tool version 11.5. The nodes (proteins) are represented in the circles, and the edges (interactions) are represented with lines.

Supplementary Document S1. Individual genomic profiles of the 28 osteosarcomas cohort. On the x-axis, the chromosomes are indicated from 1 to 22 and X-Y. The y-axis contains the $\log_2$ sample/reference ratio range for CNA calls (values ≥ 0.25 were considered gains, ≤ -0.25 losses, ≥ 1.2 high copy gains, and ≤ -1.2 homozygous copy losses). Images were obtained from Nexus Copy Number software version 9.0.
