# Supplementary figures and images for: Analysis of the Mutational Landscape of Osteosarcomas Identifies Genes Related to Metastasis and Prognosis and Disrupted Biological Pathways of Immune Response and Bone Development
Source: Int J Mol Sci. 2023 Jun 21;24(13):10463. doi: 10.3390/ijms241310463 (PMC10342084; doi:10.3390/ijms241310463)

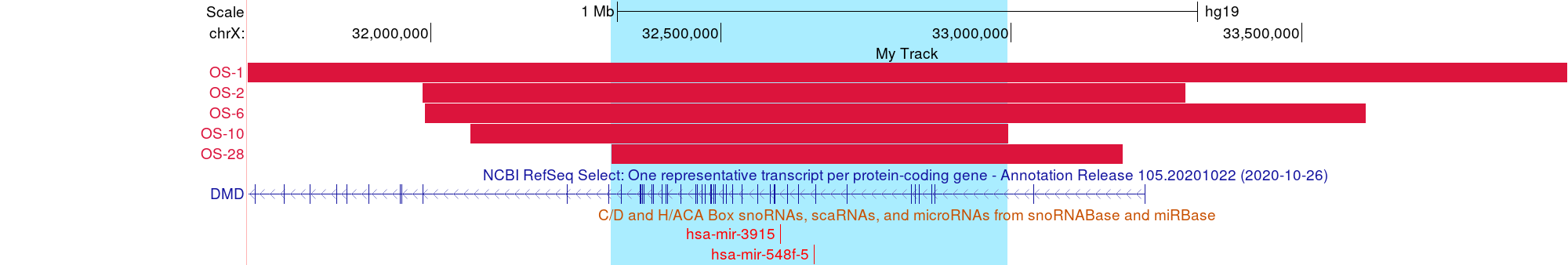

Supplement: Supplementary file 1 [file ijms-24-10463-s001.zip › Supplementary Figure S1.png]

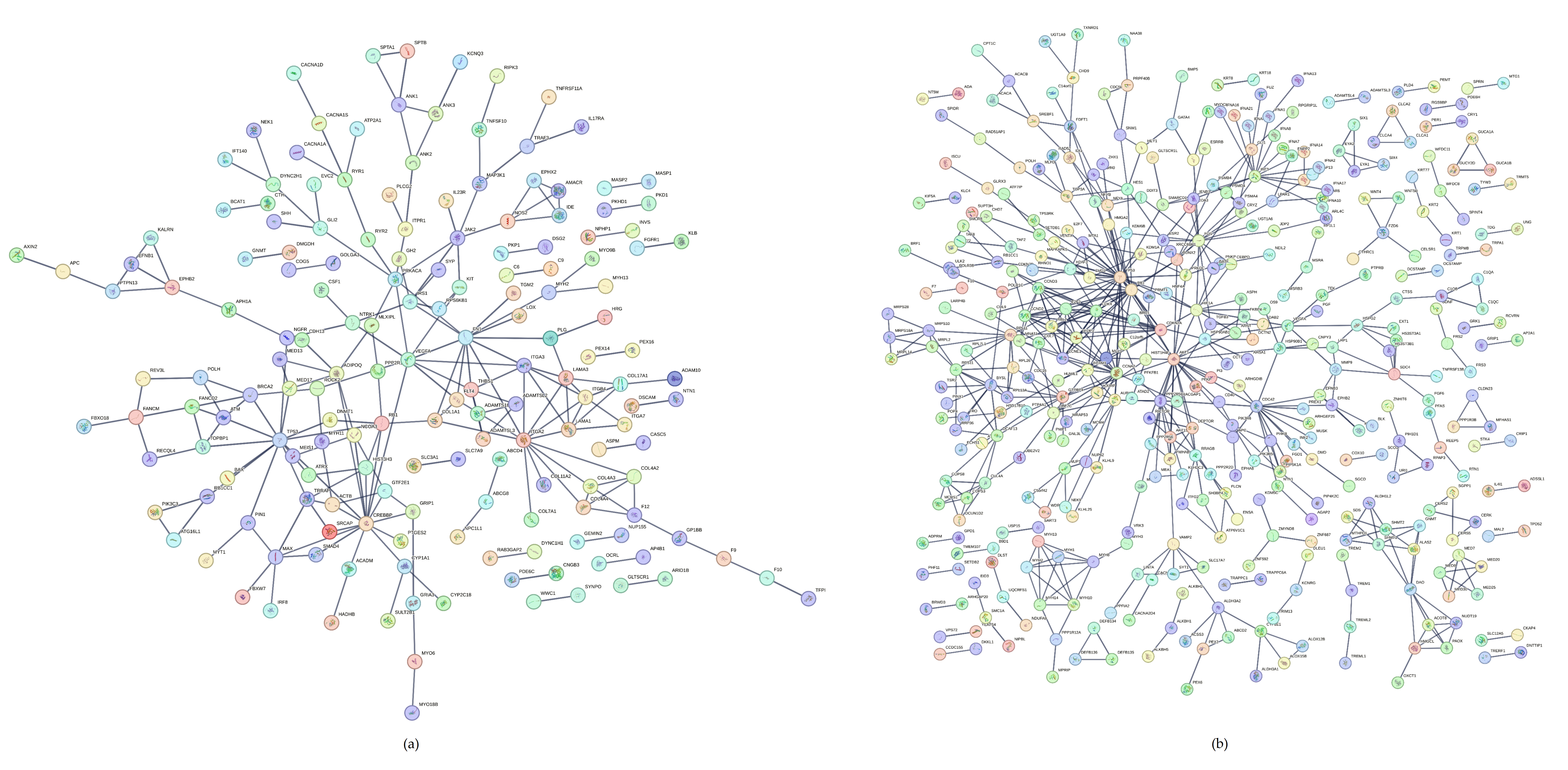

Supplement: Supplementary file 1 [file ijms-24-10463-s001.zip › Supplementary Figure S2.png]

OS-1

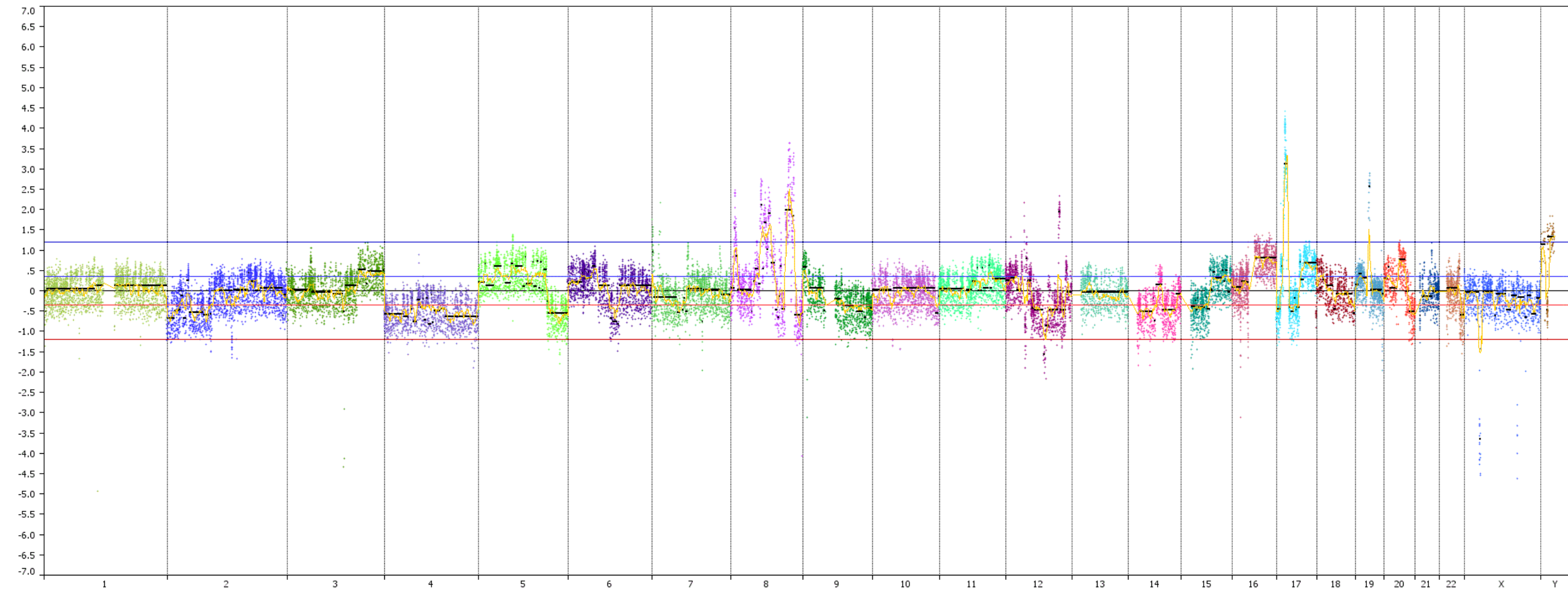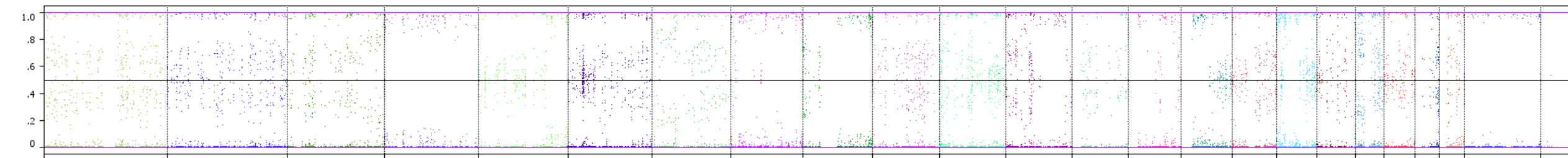

OS-2

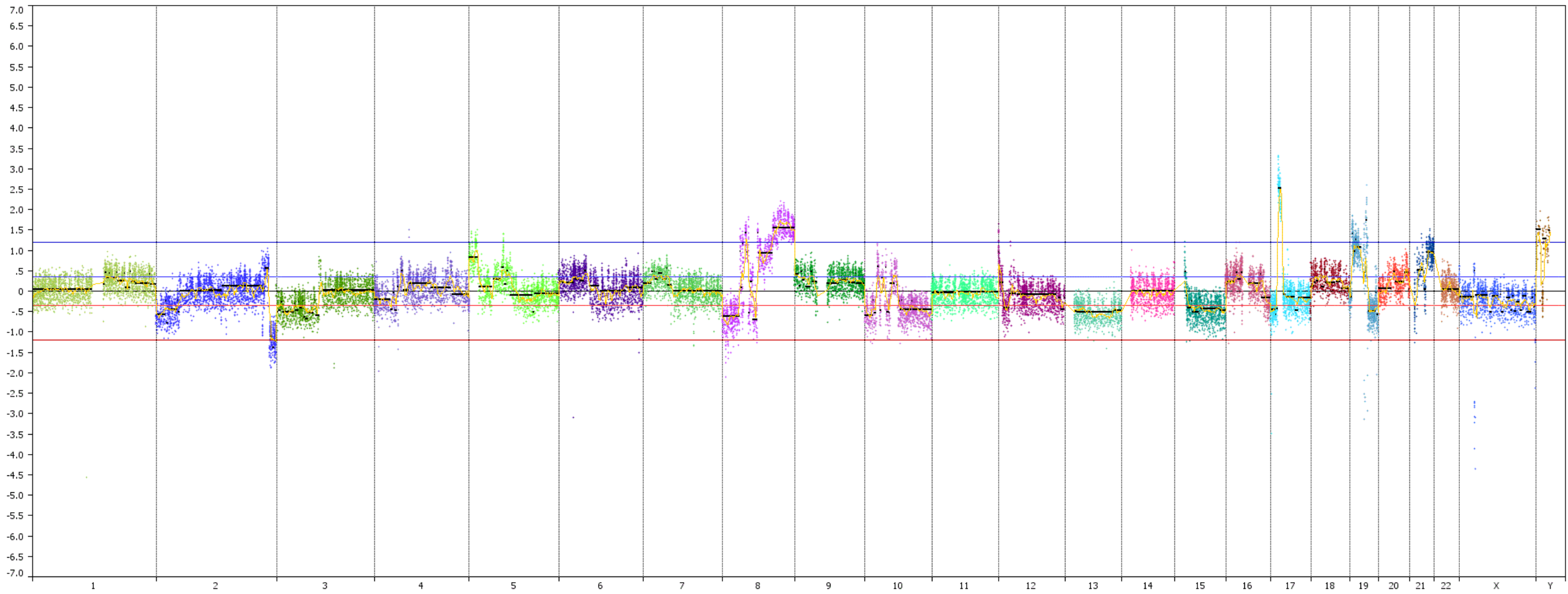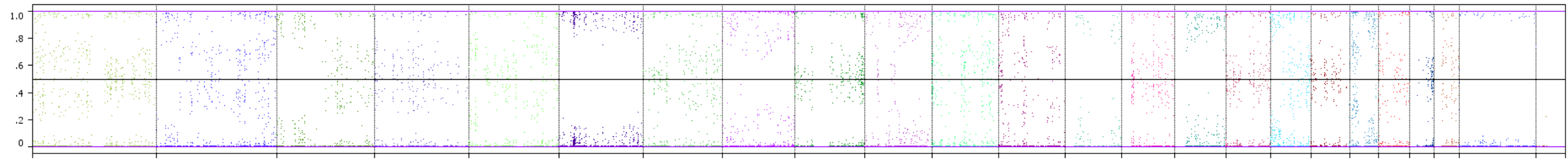

OS-3

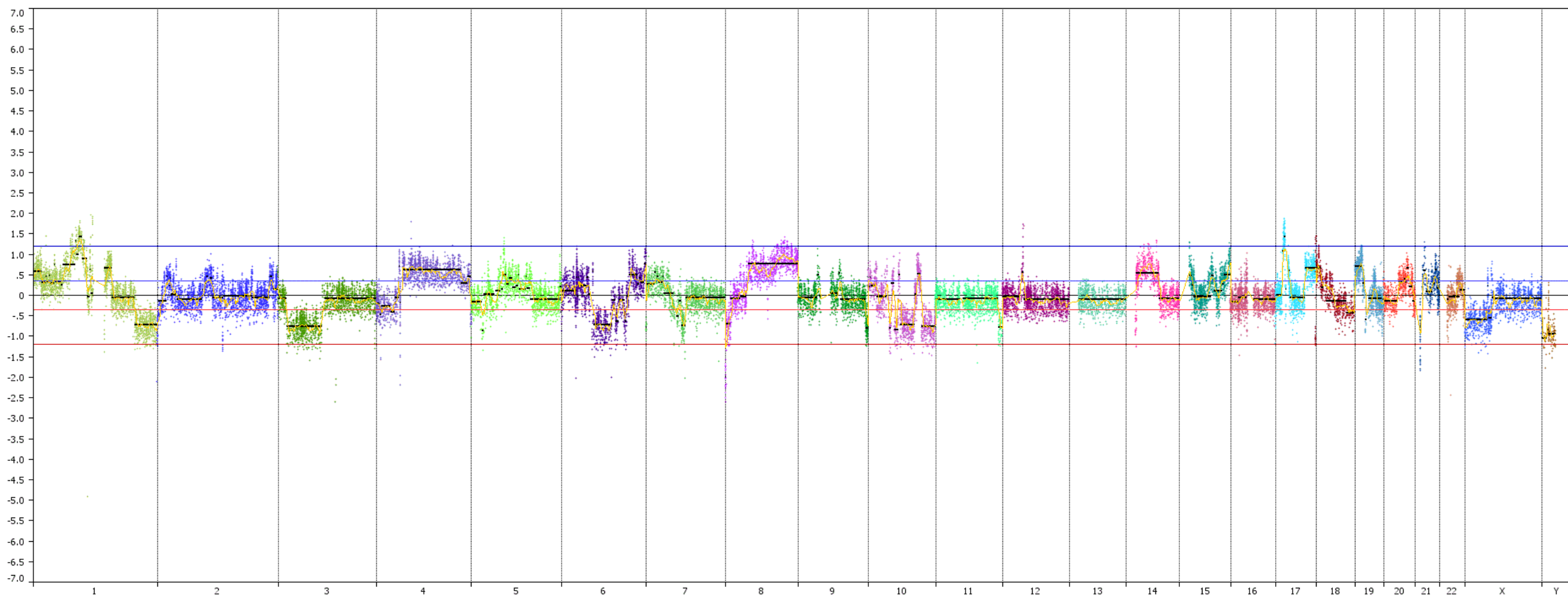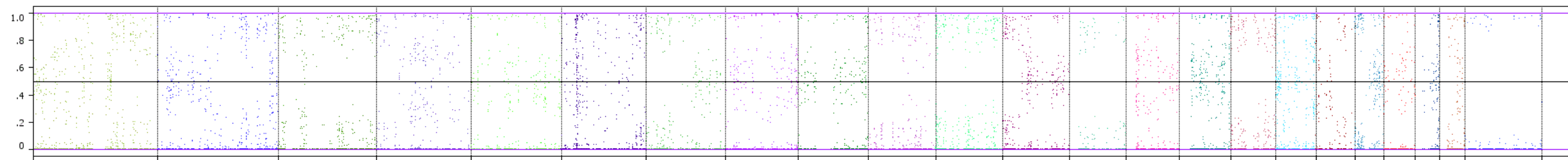

OS-4

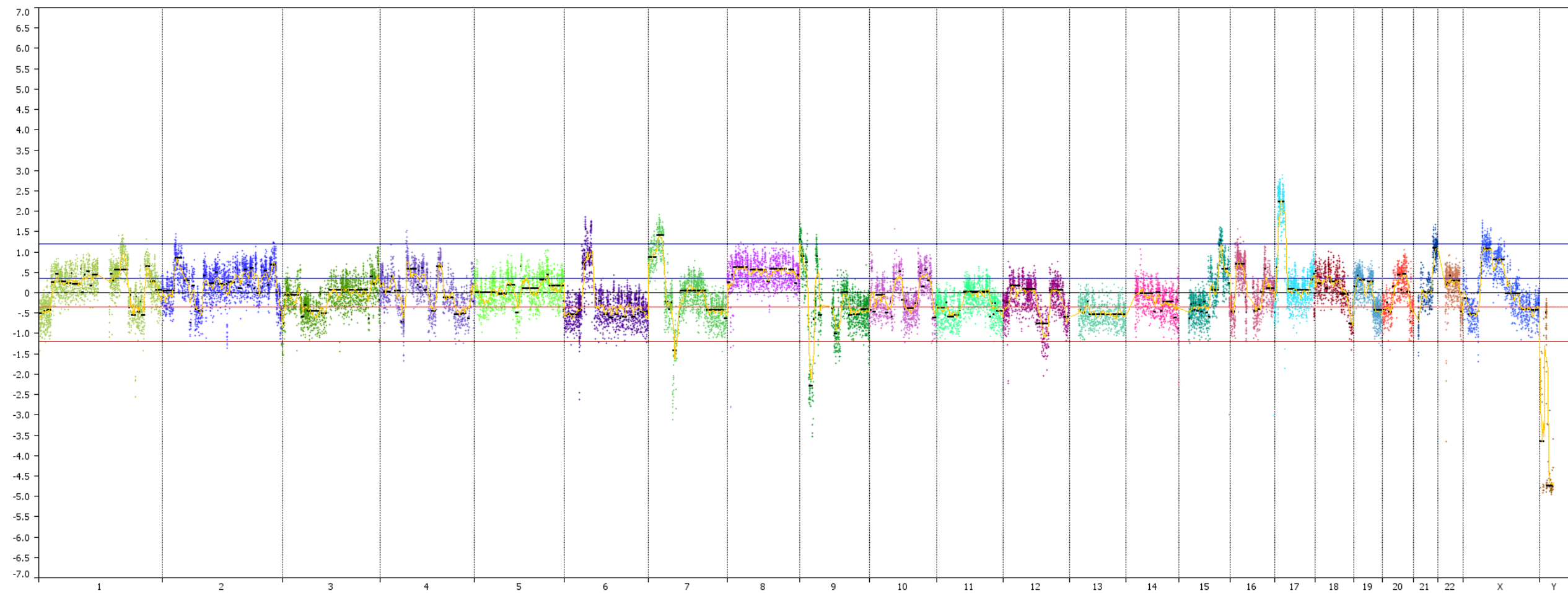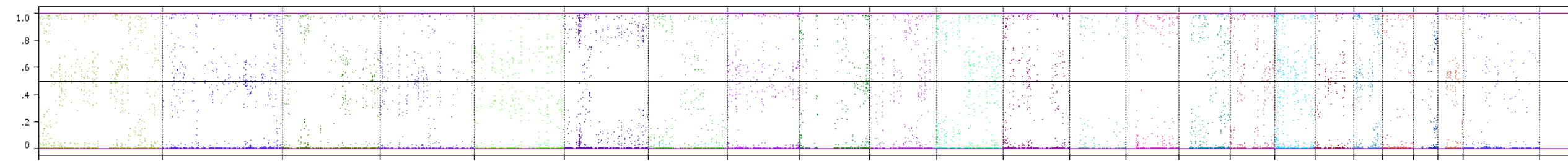

OS-5

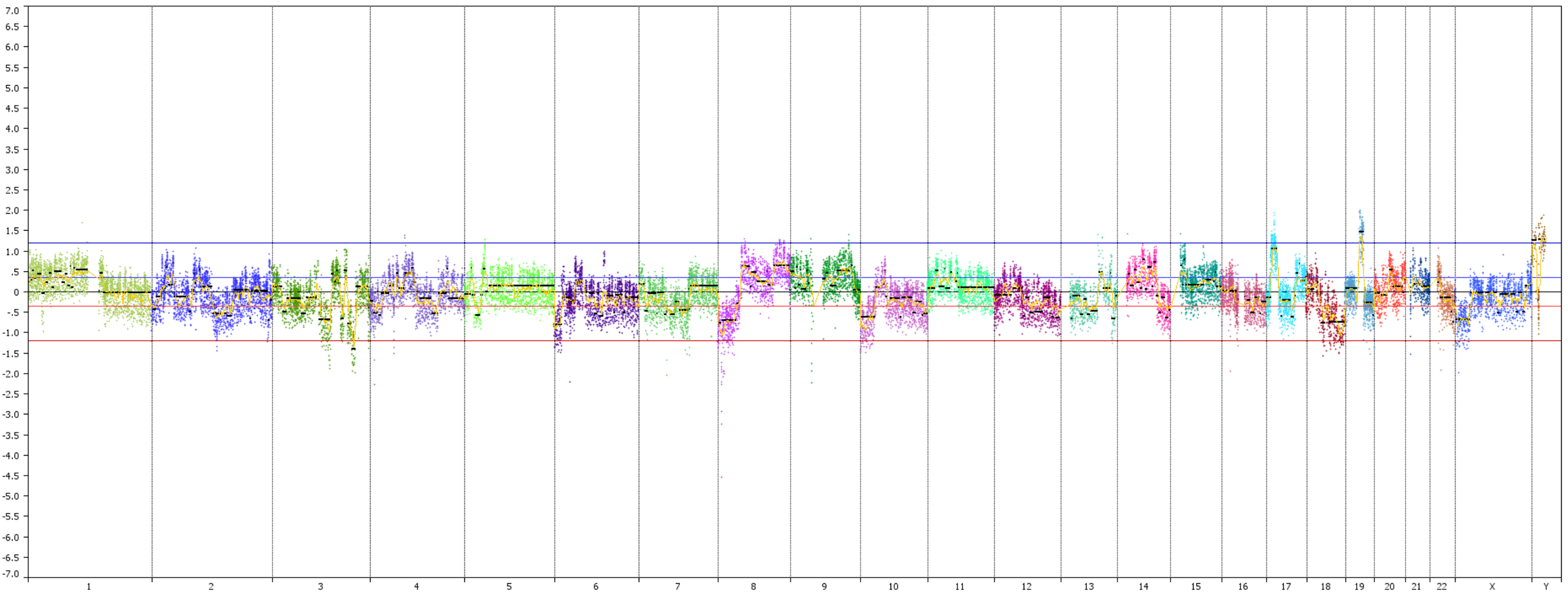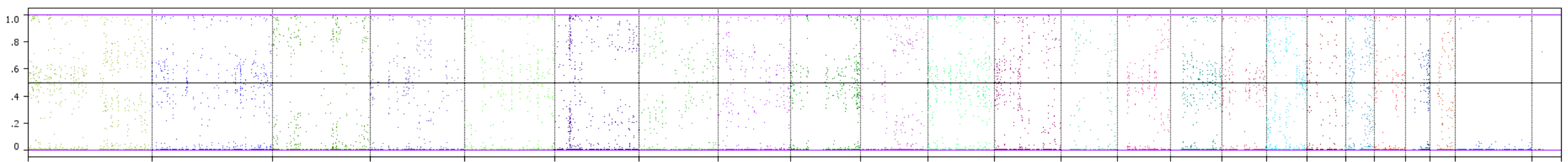

OS-6

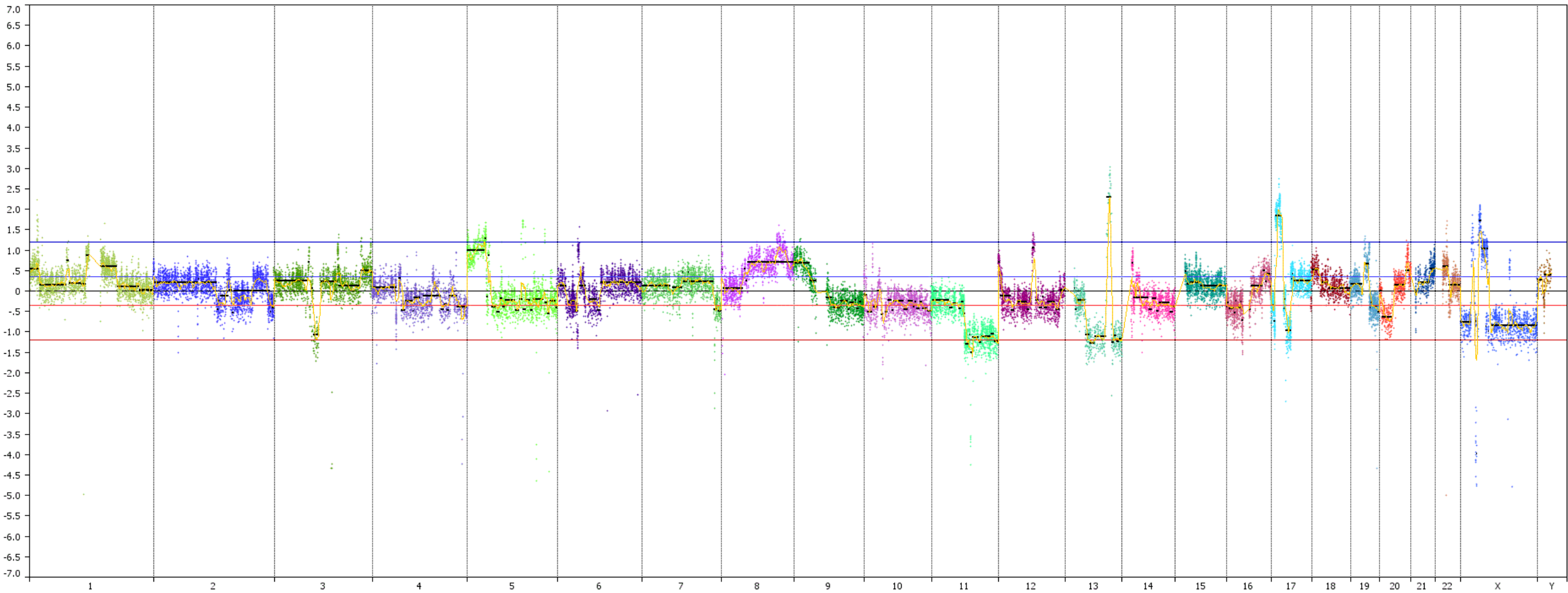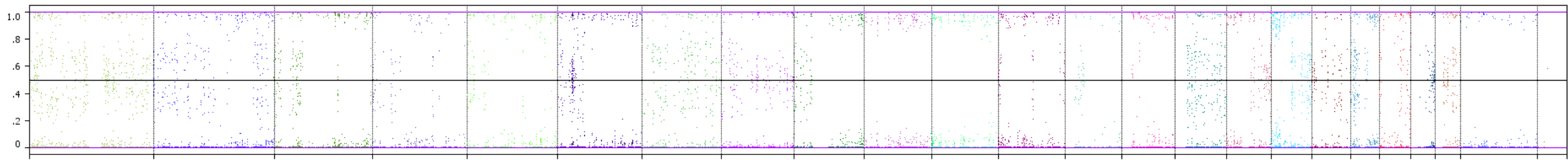

OS-7

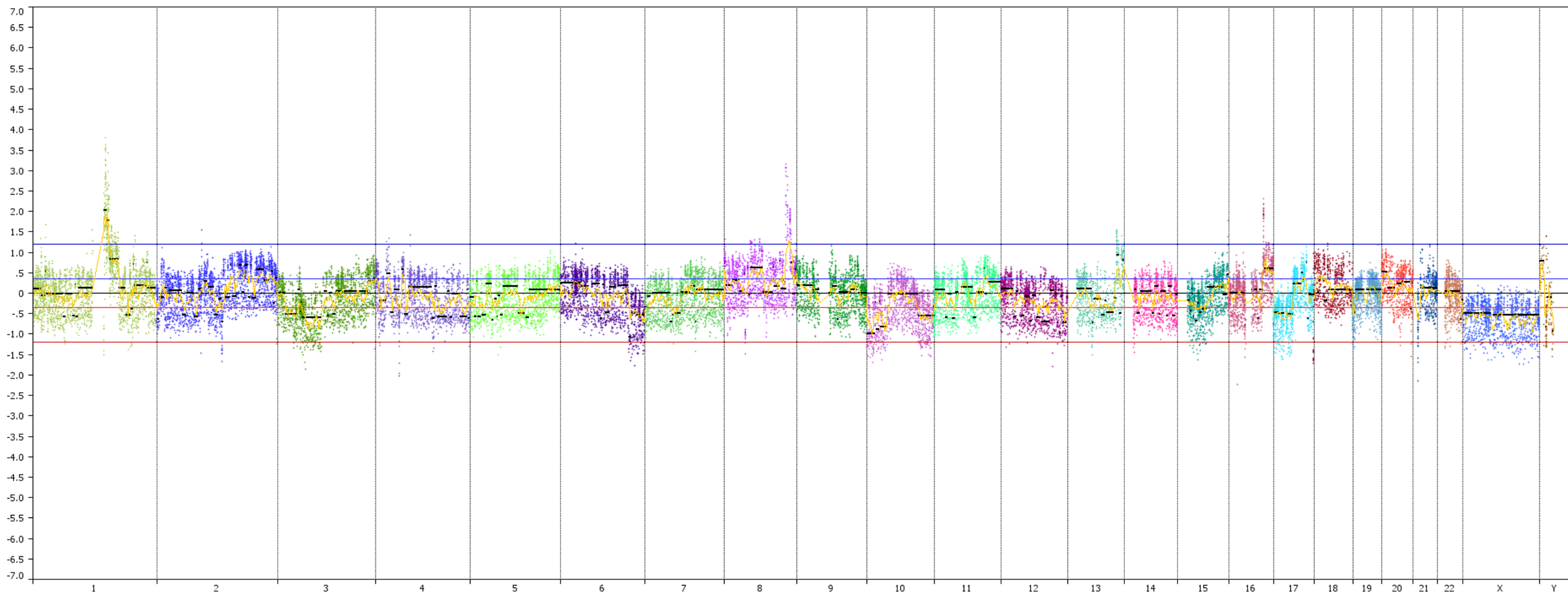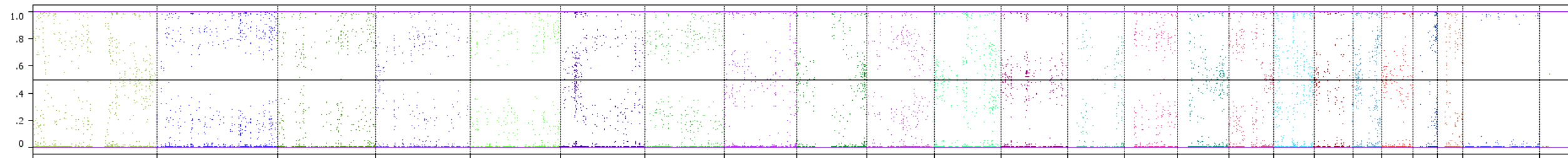

OS-8

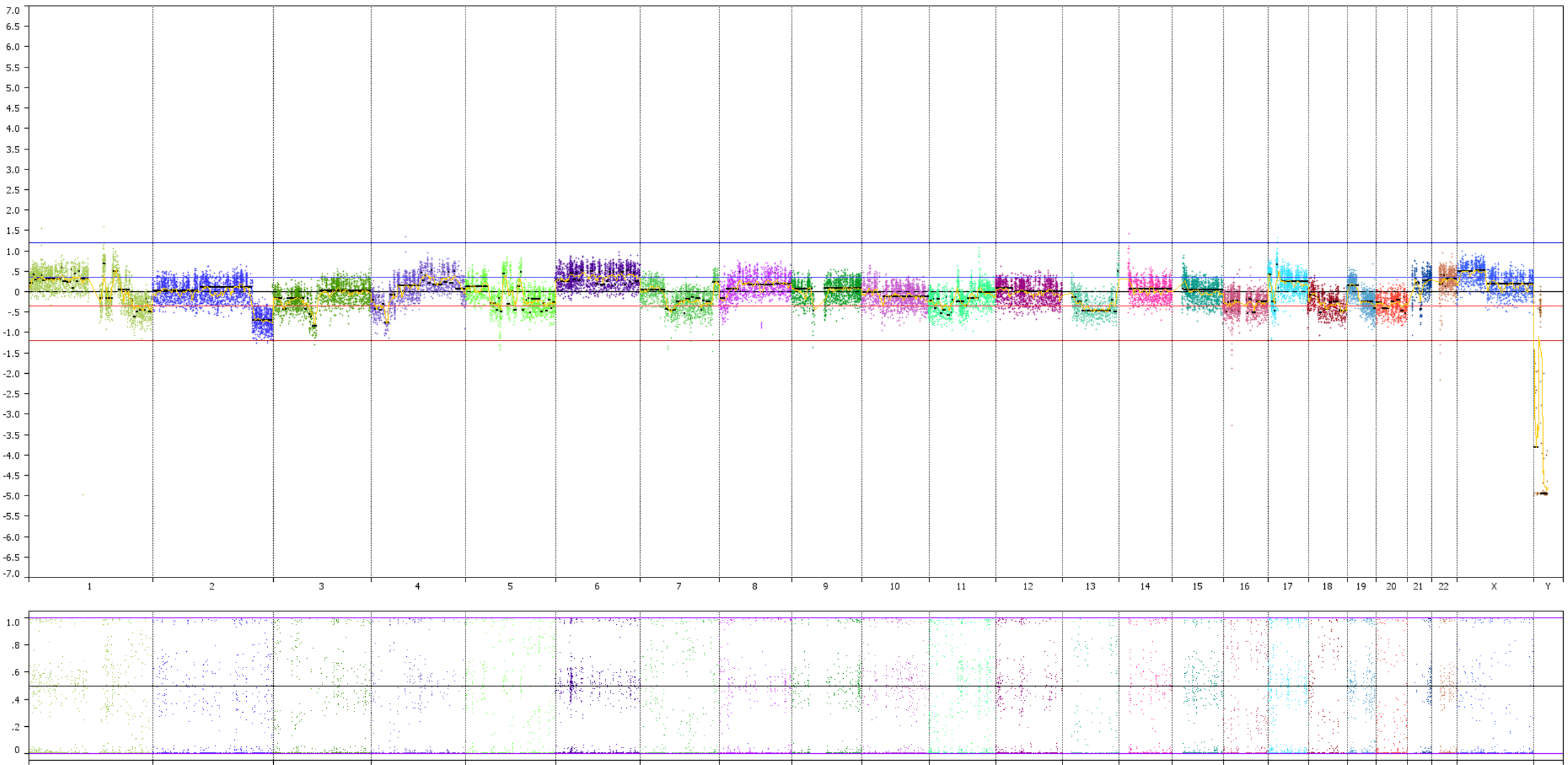

OS-9

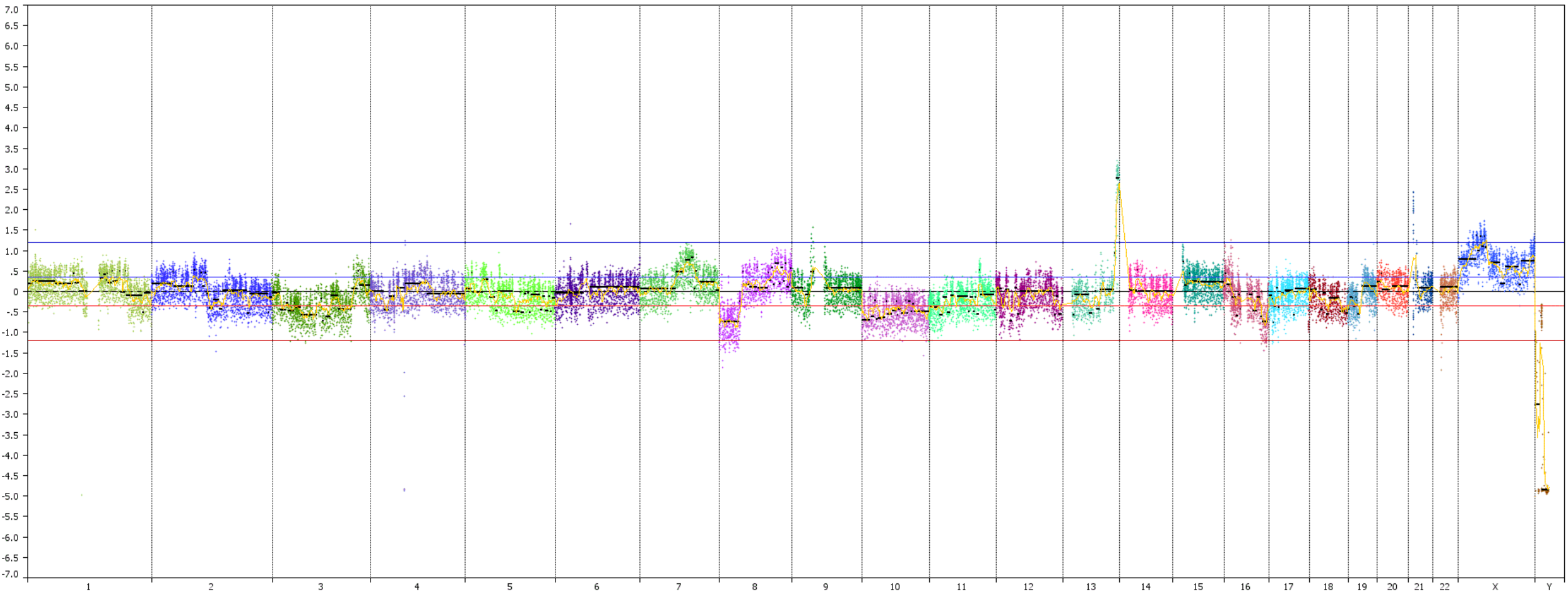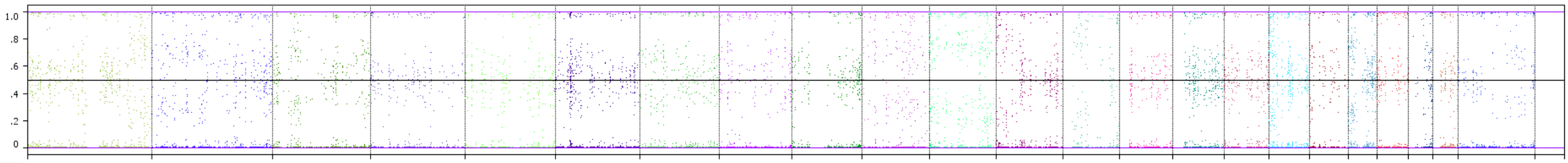

OS-10

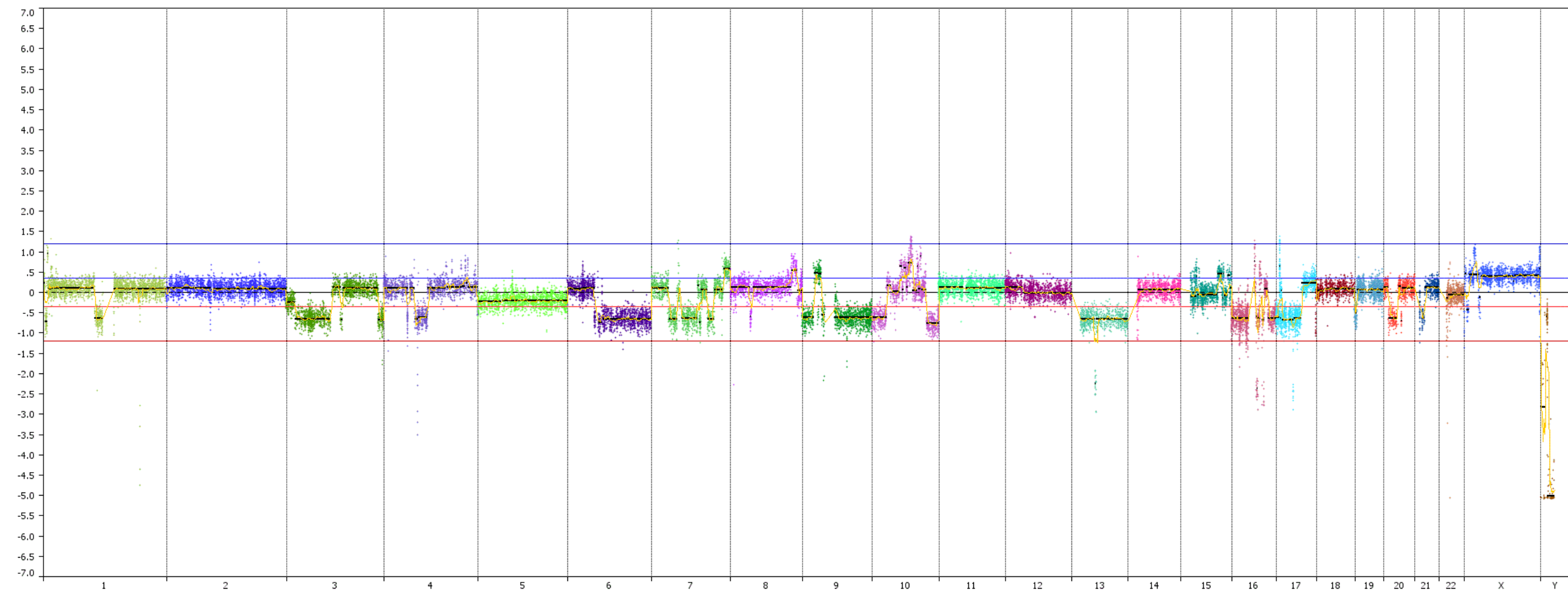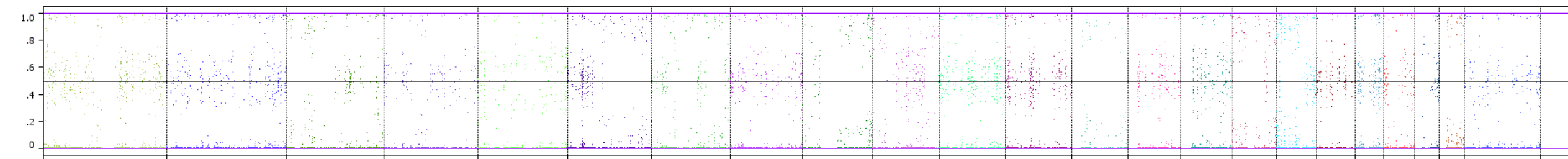

OS-11

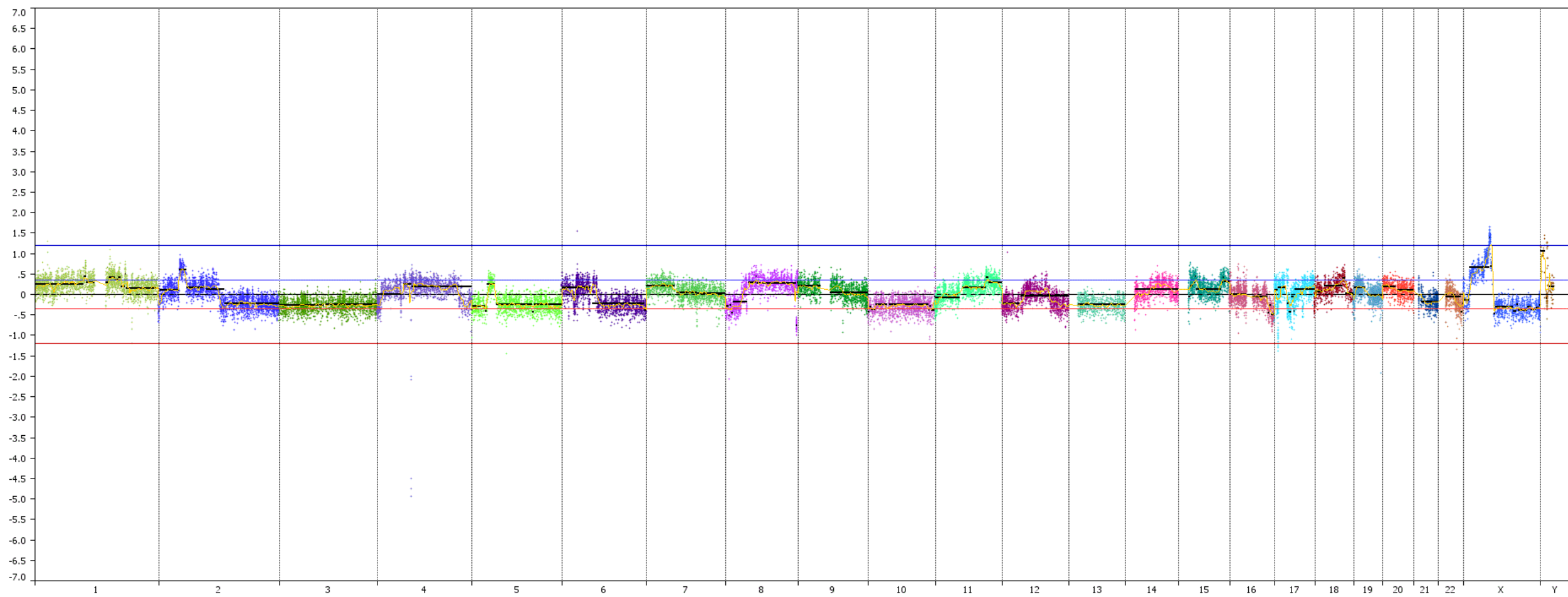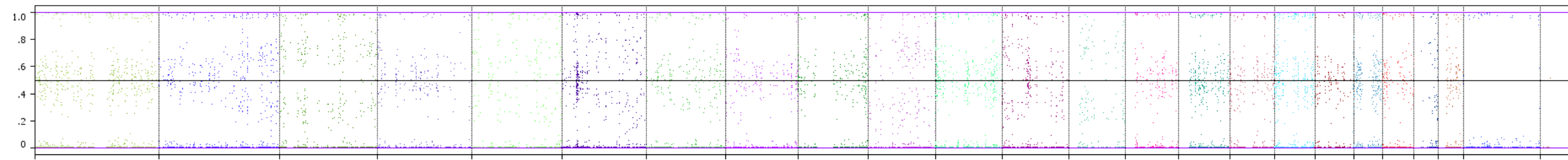

OS-12

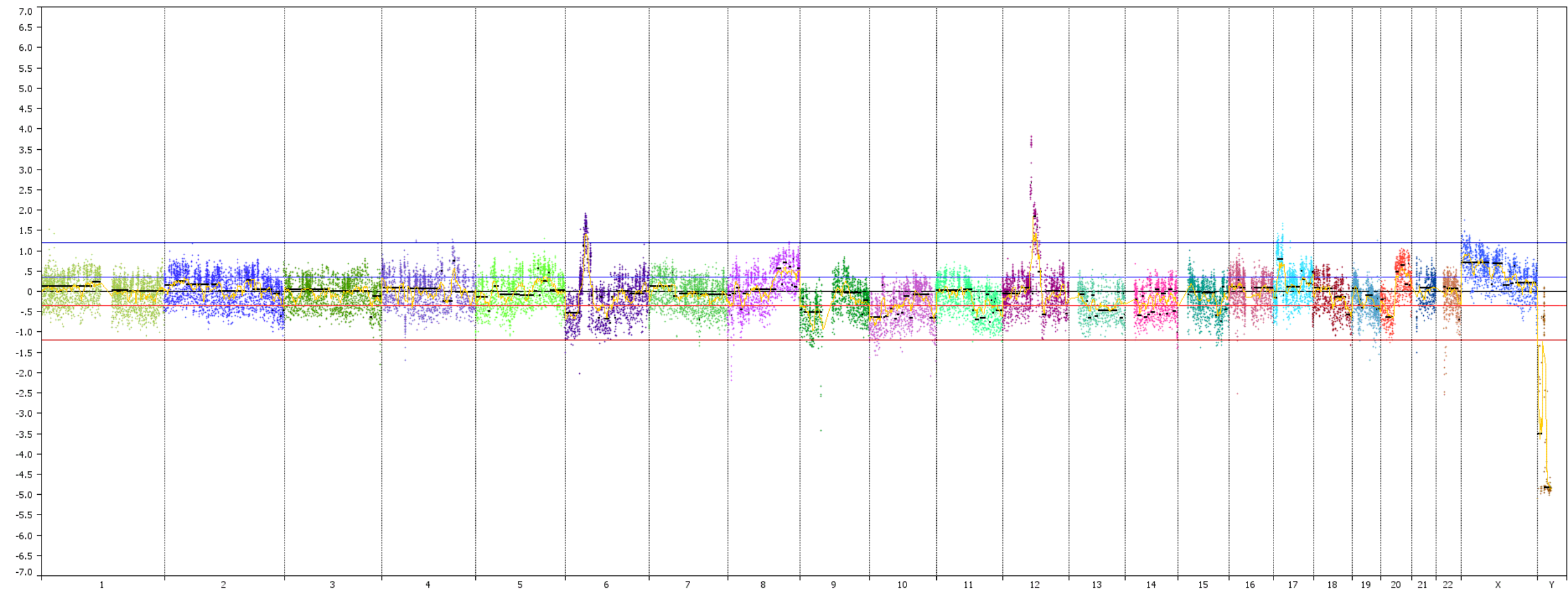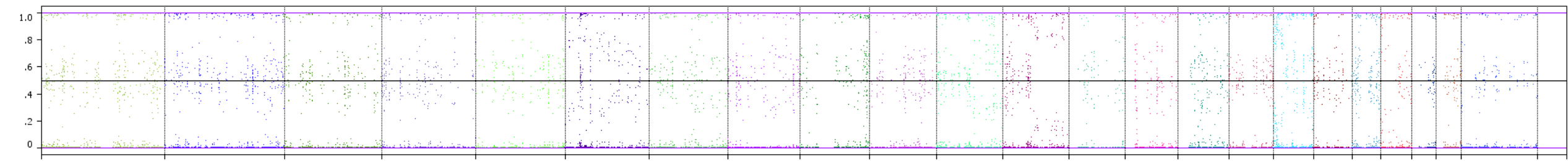

OS-13

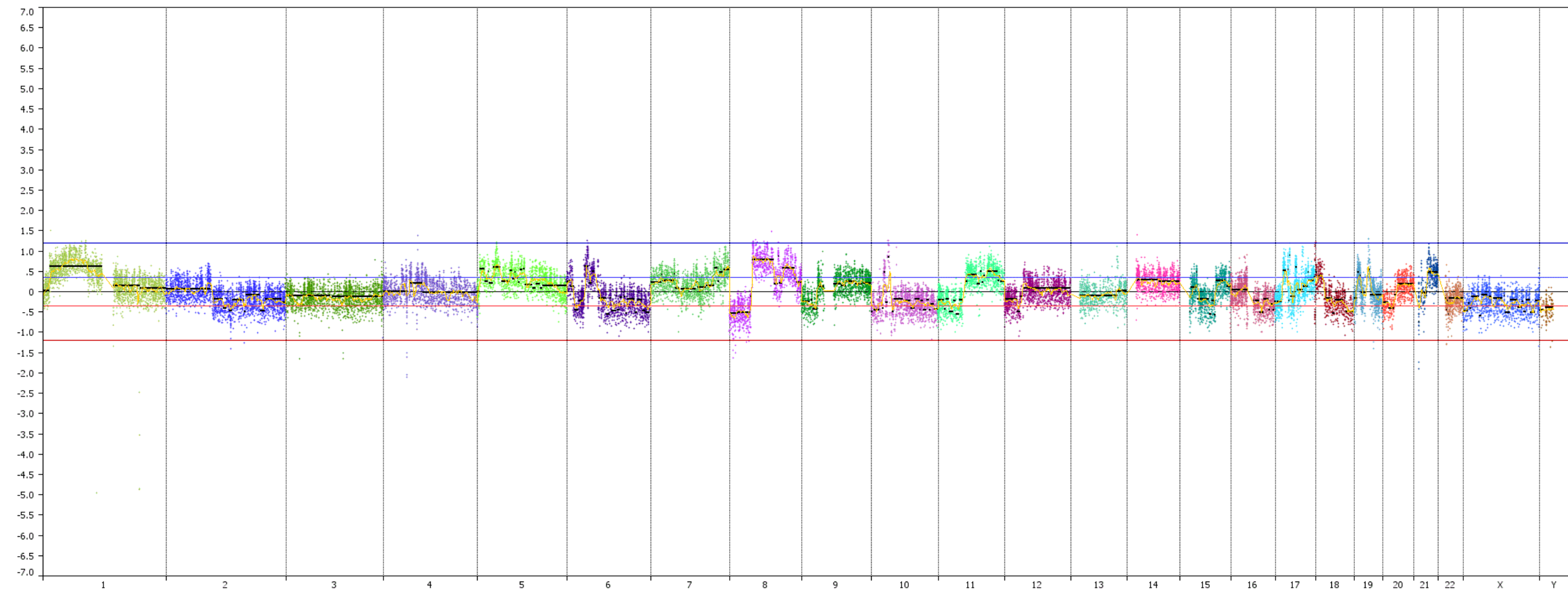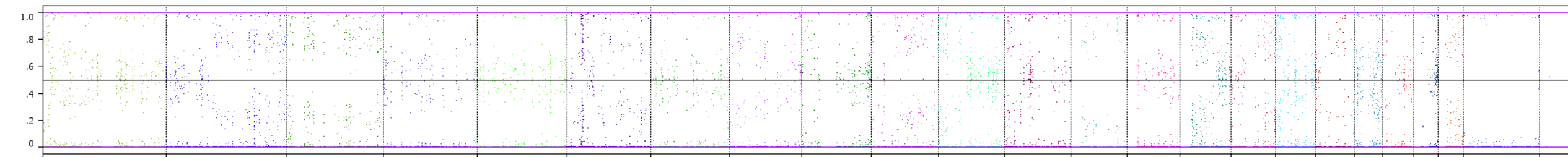

OS-14

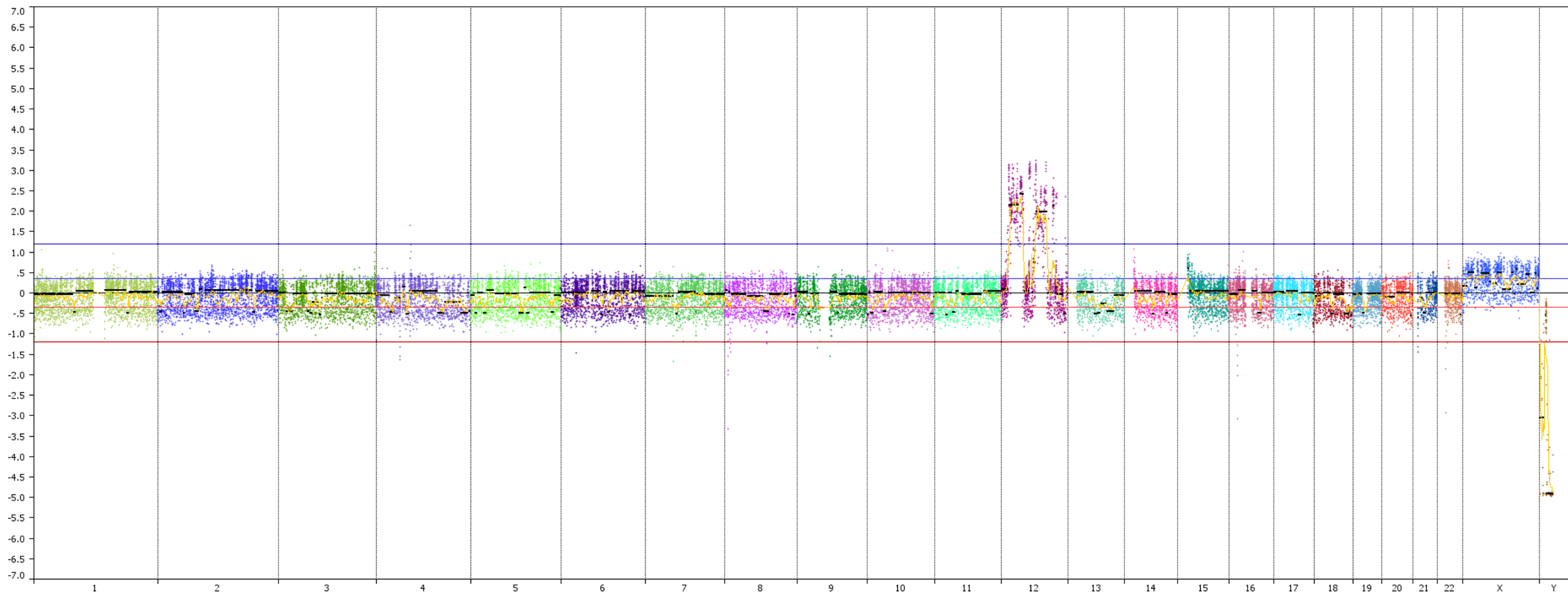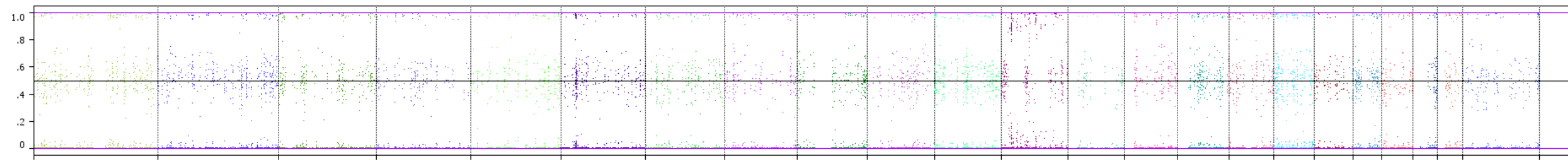

OS-15

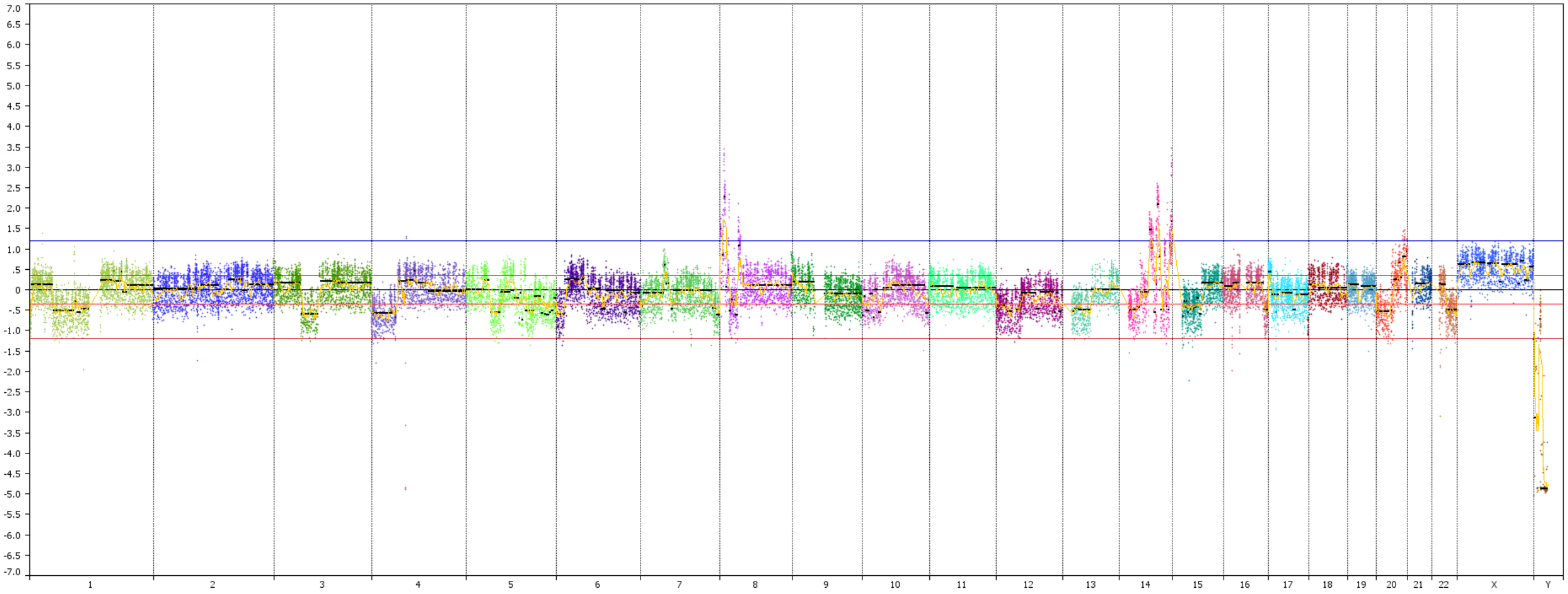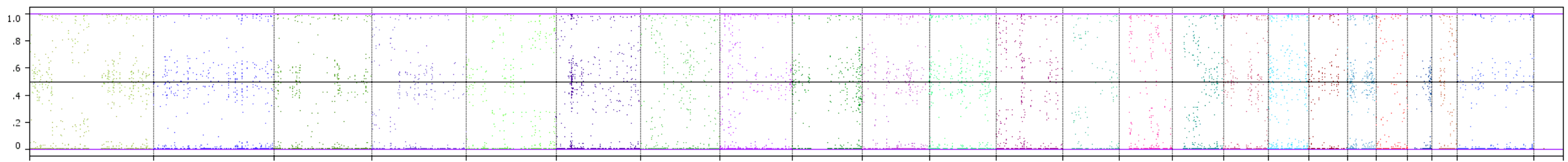

OS-16

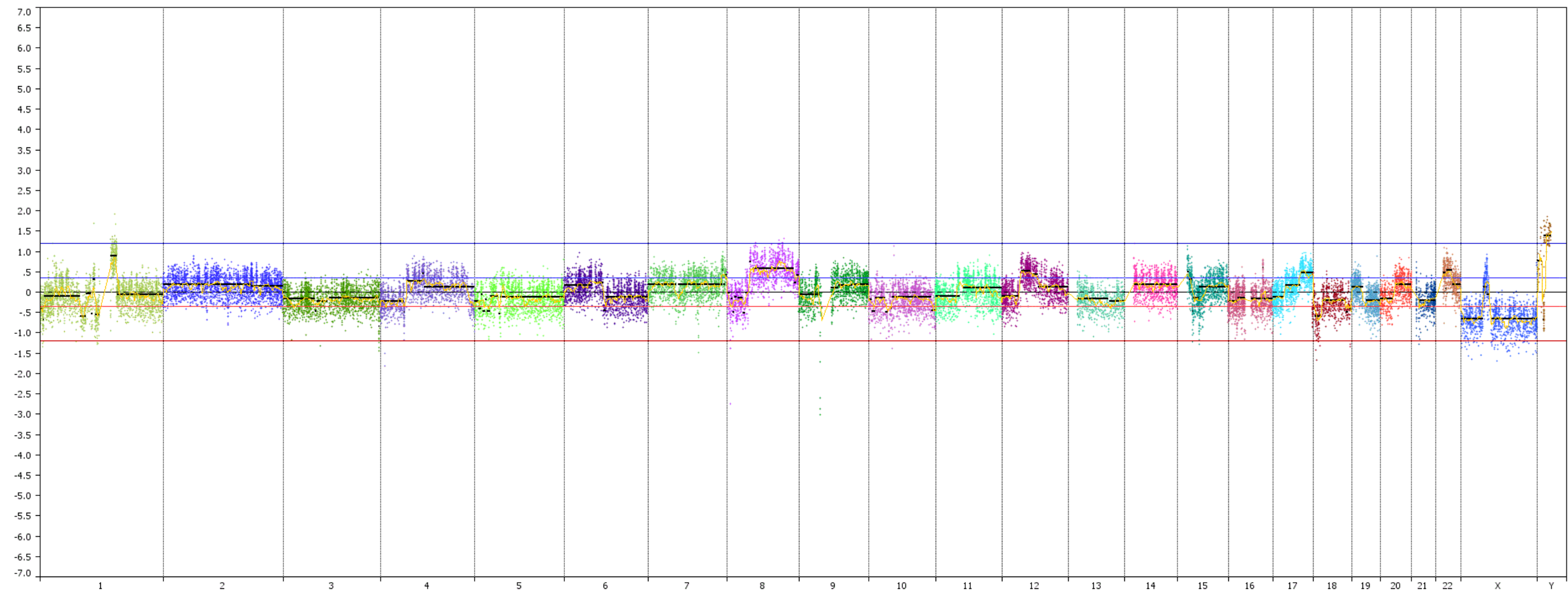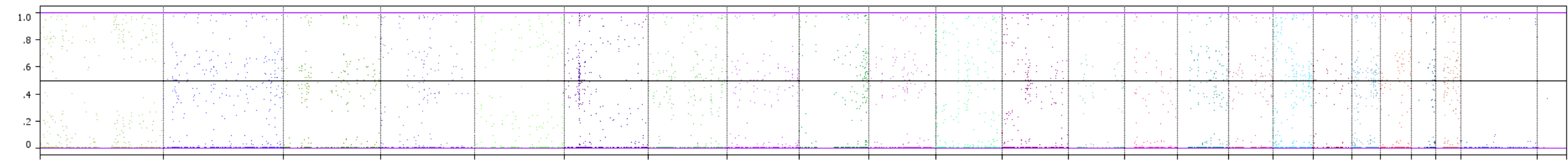



OS-18

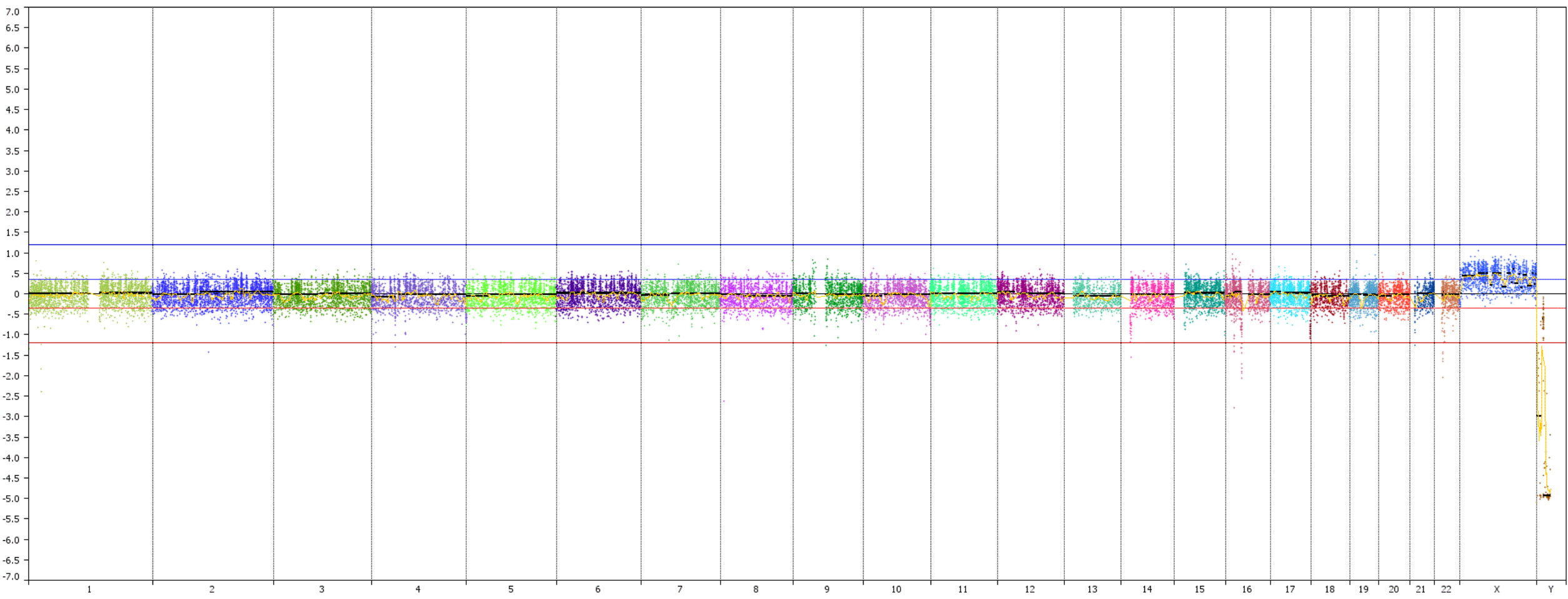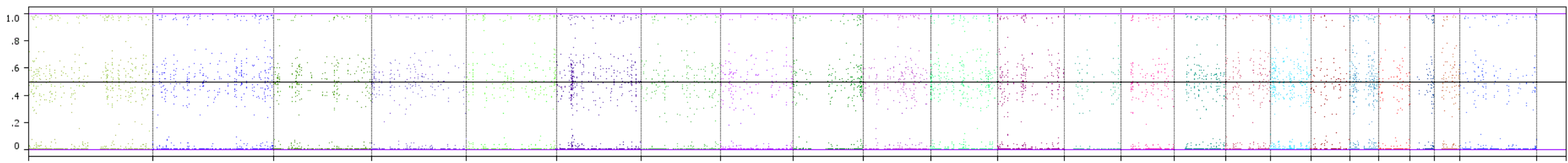

OS-19

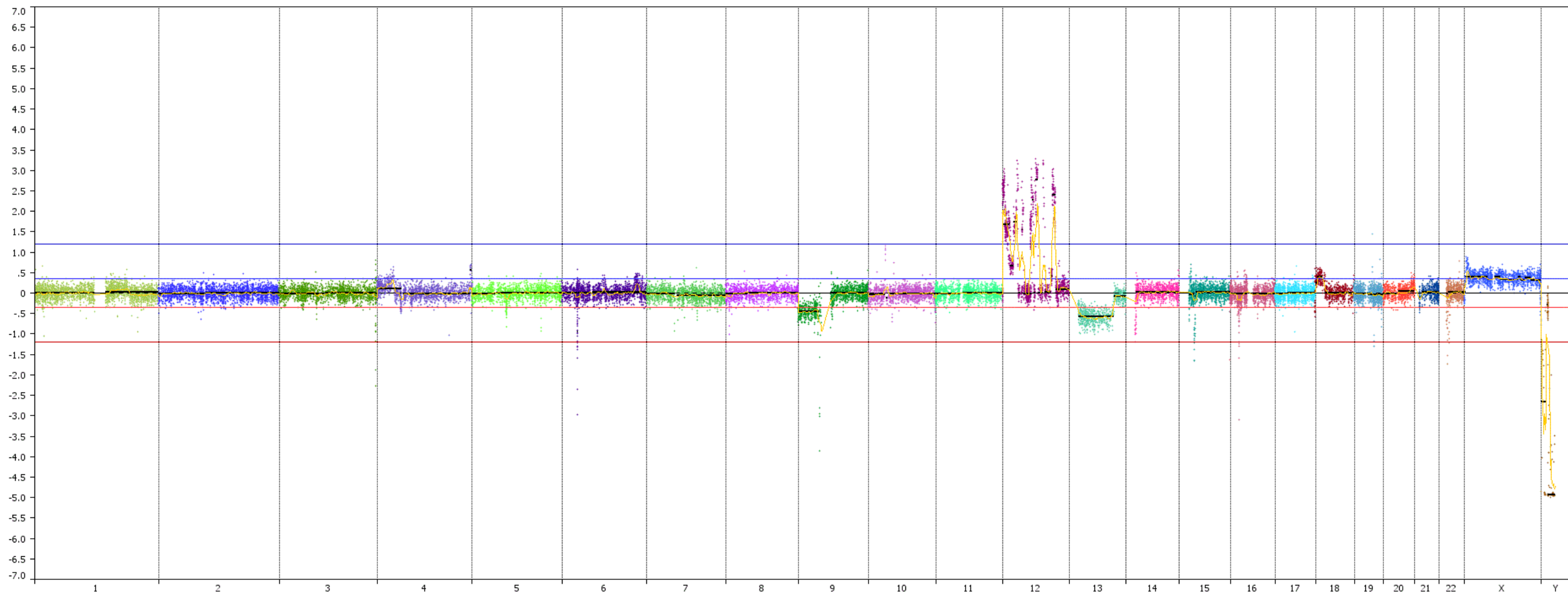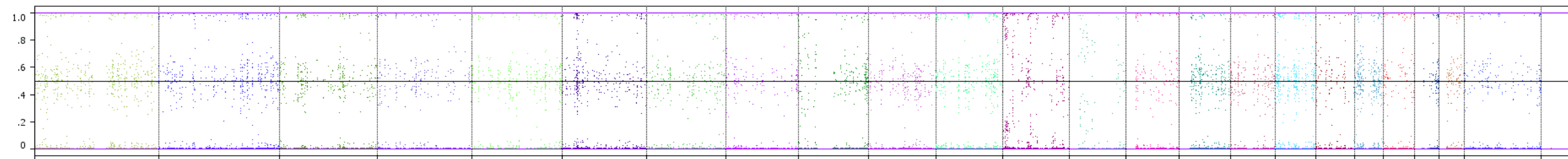

OS-20

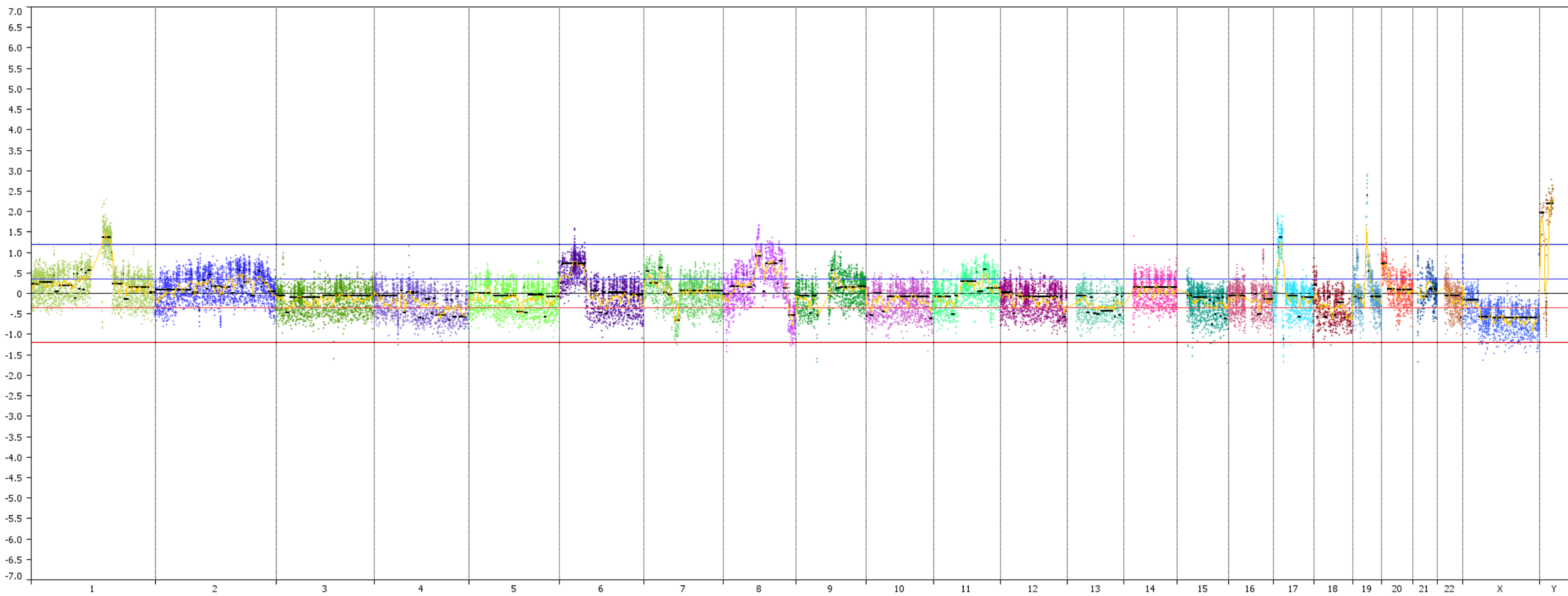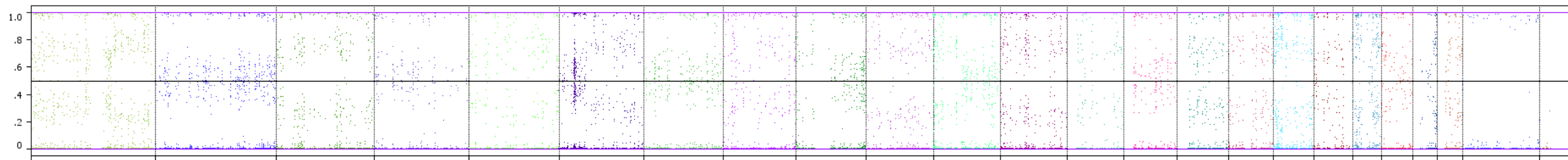

OS-21

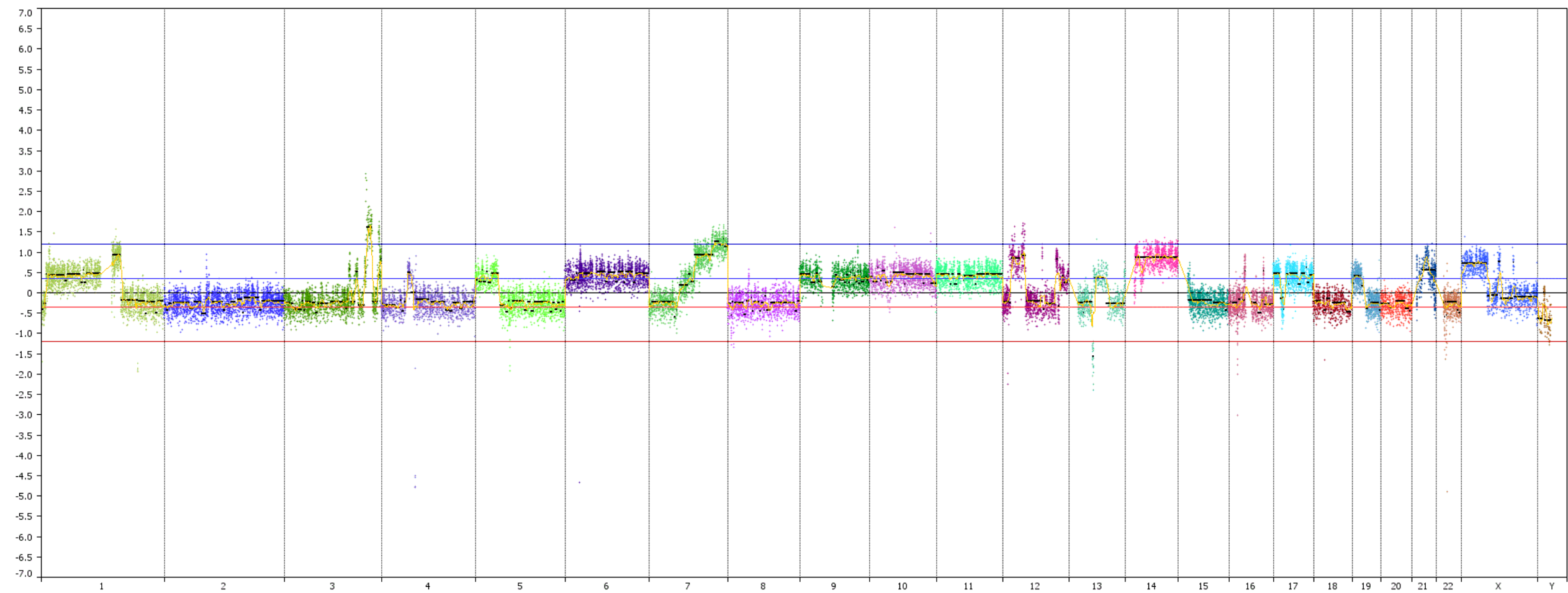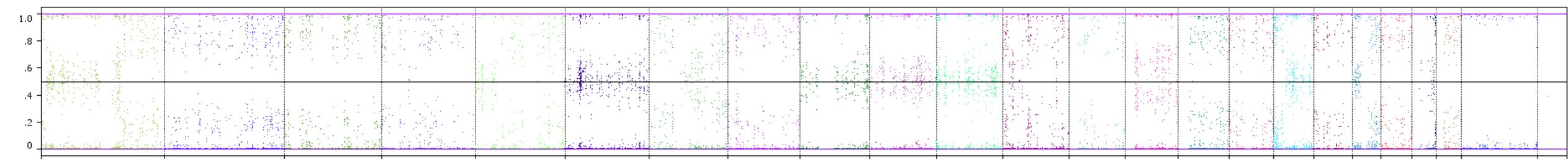

OS-22

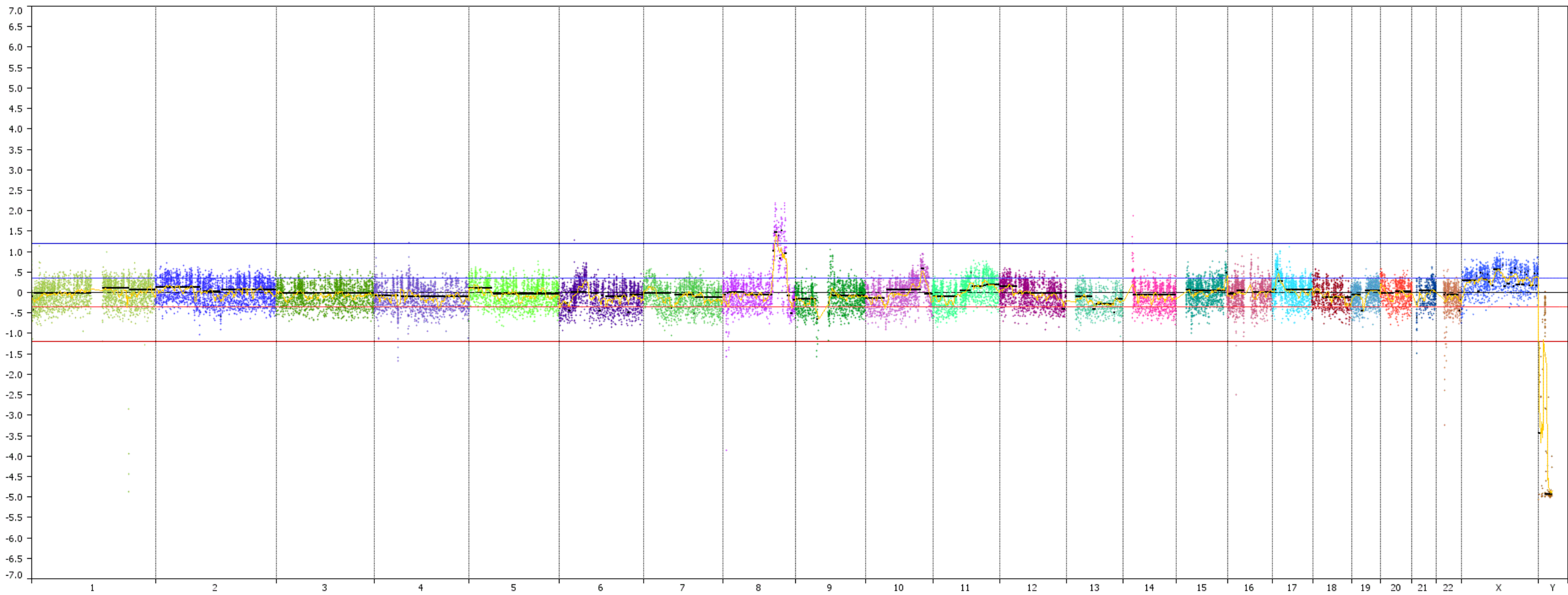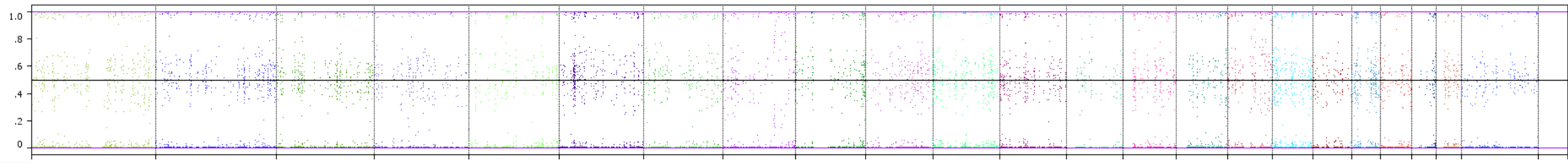

OS-23

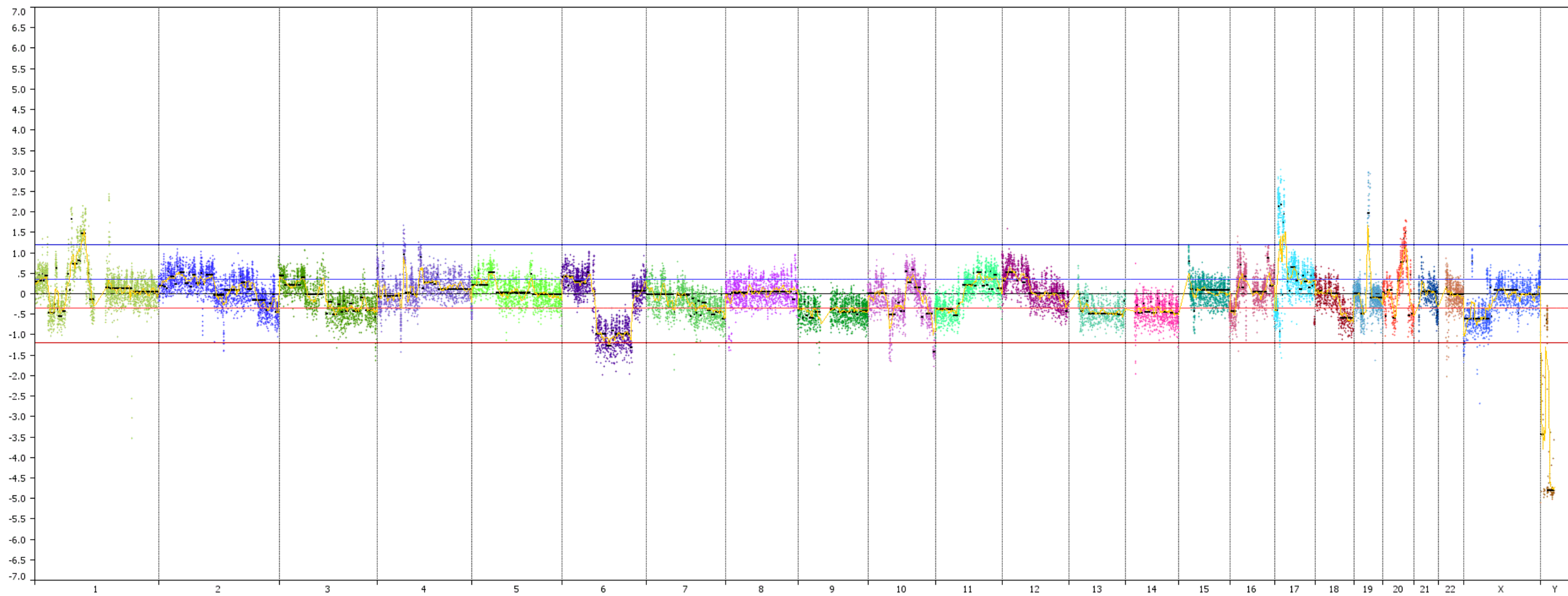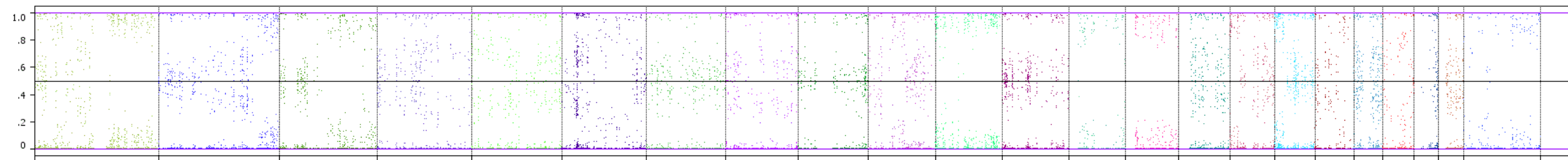

OS-24

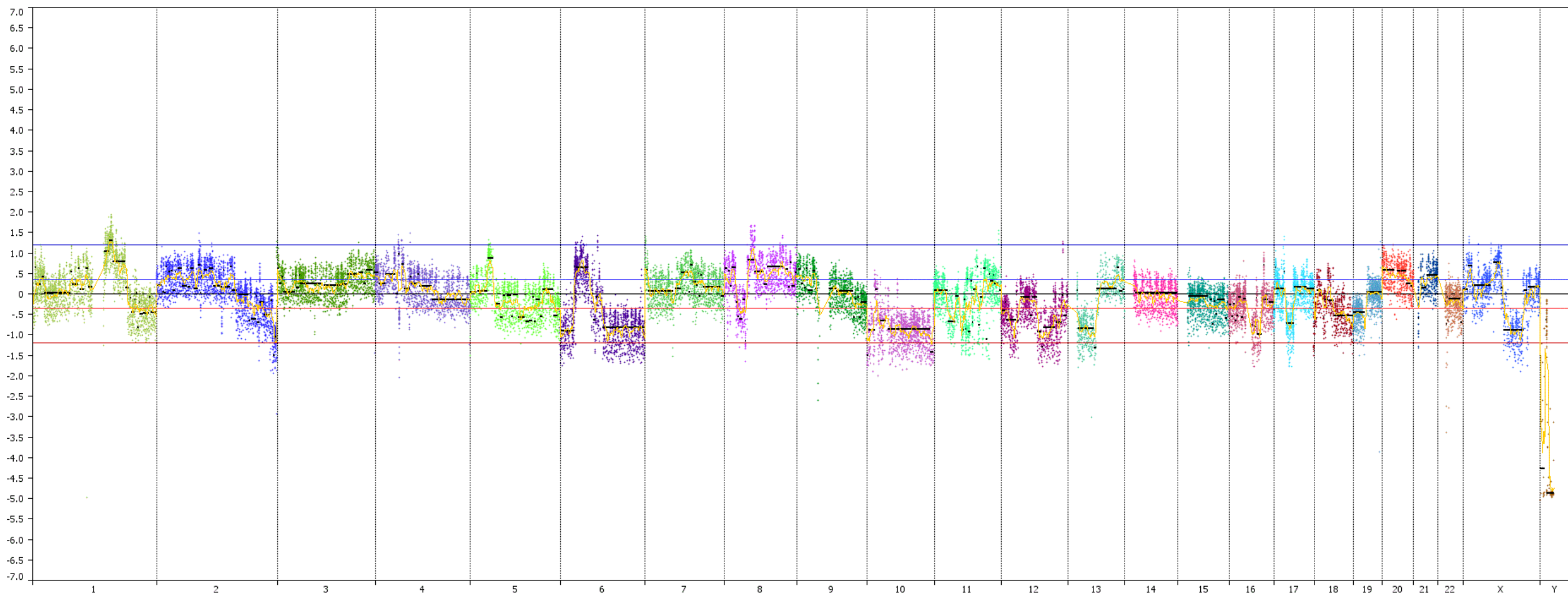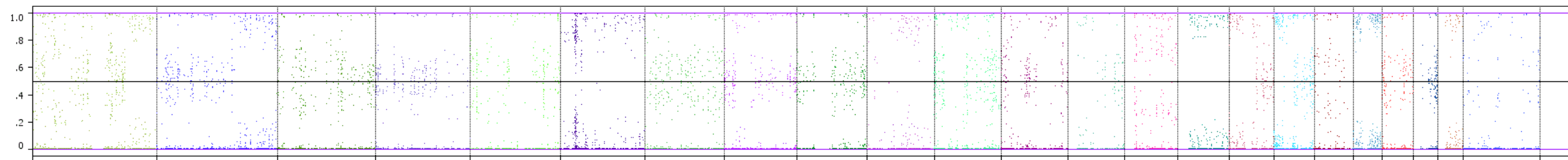

OS-25

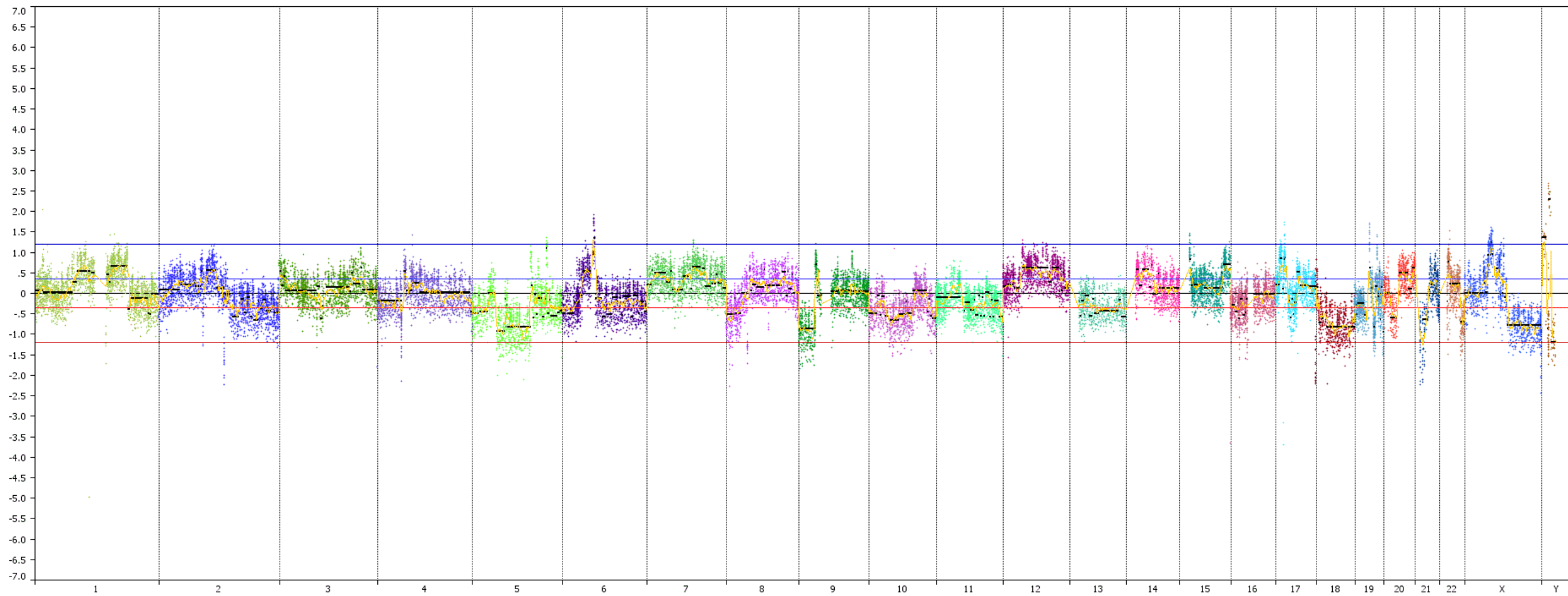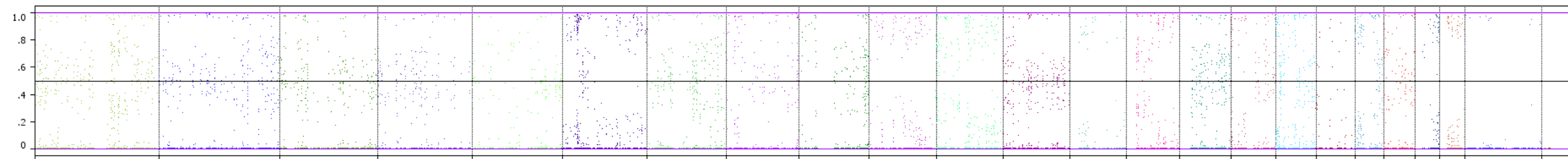

OS-26

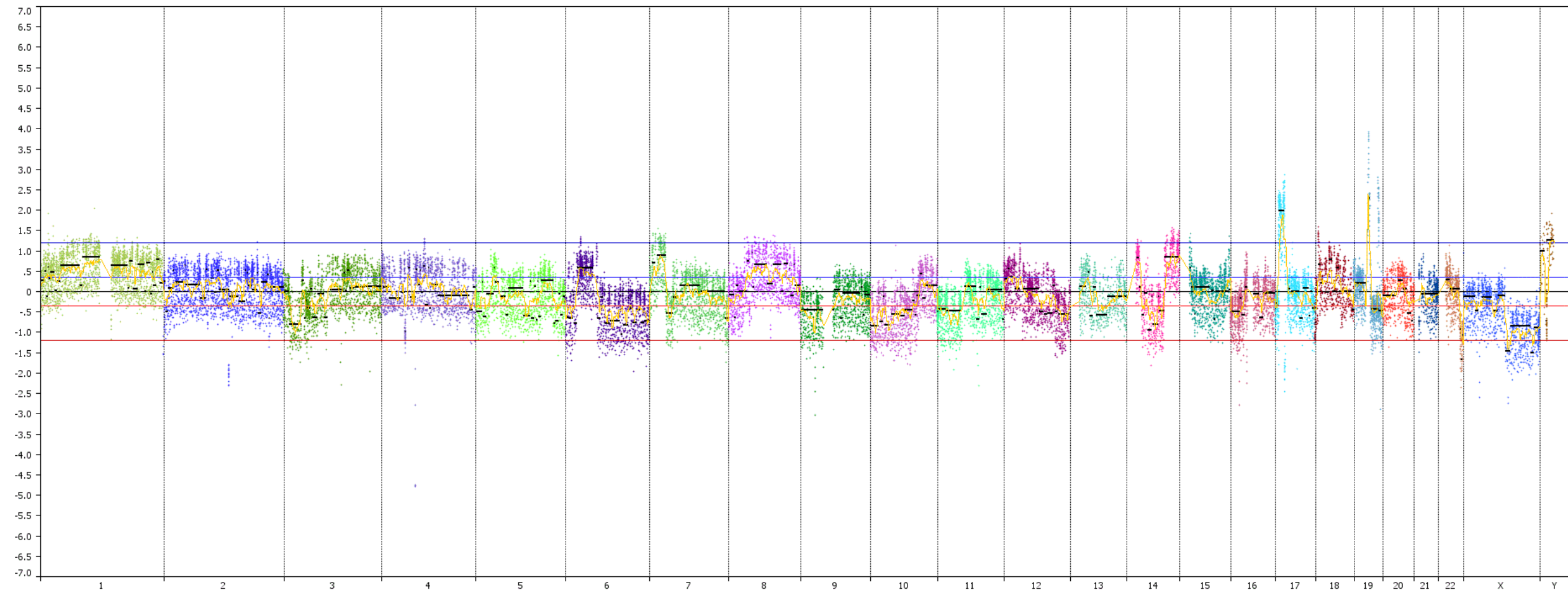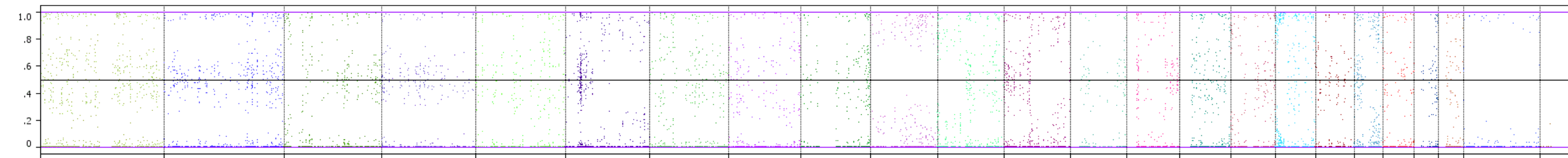

OS-27

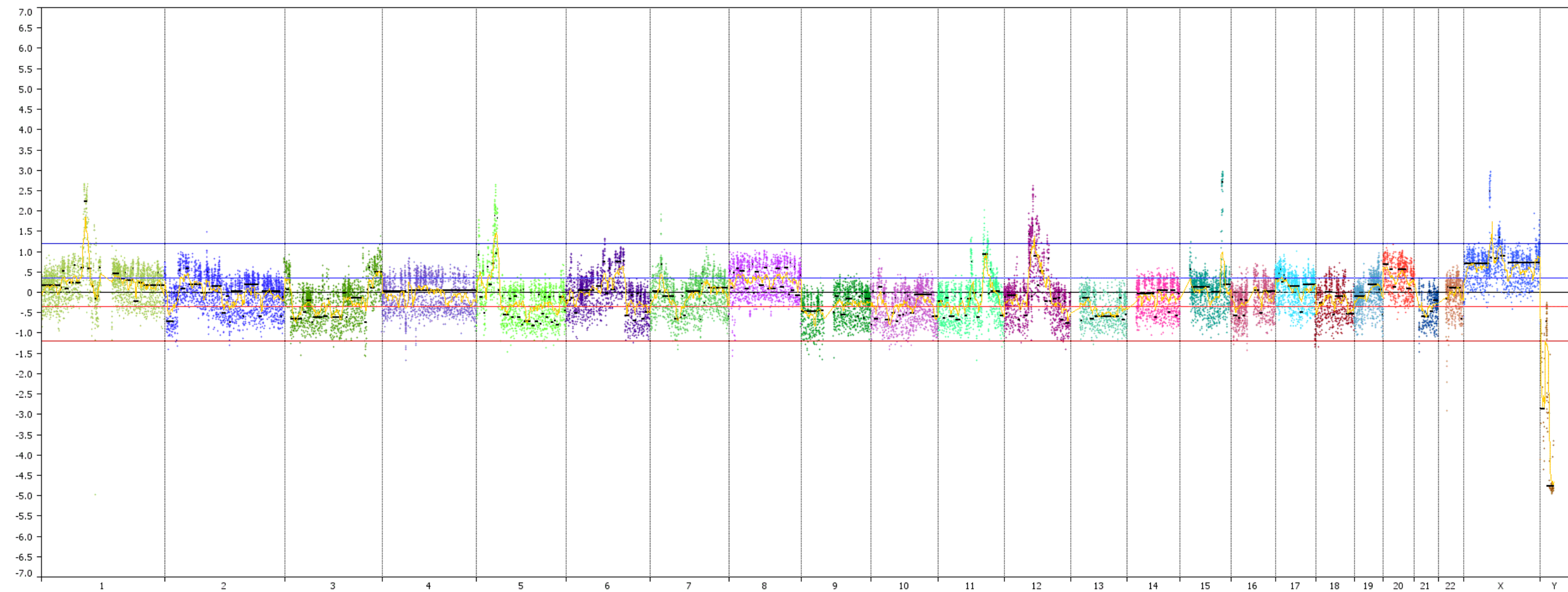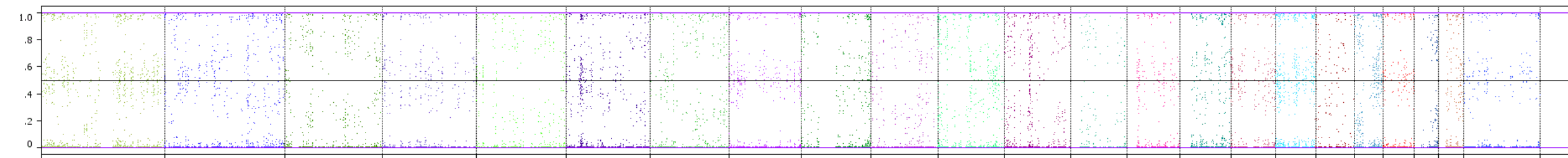

OS-28

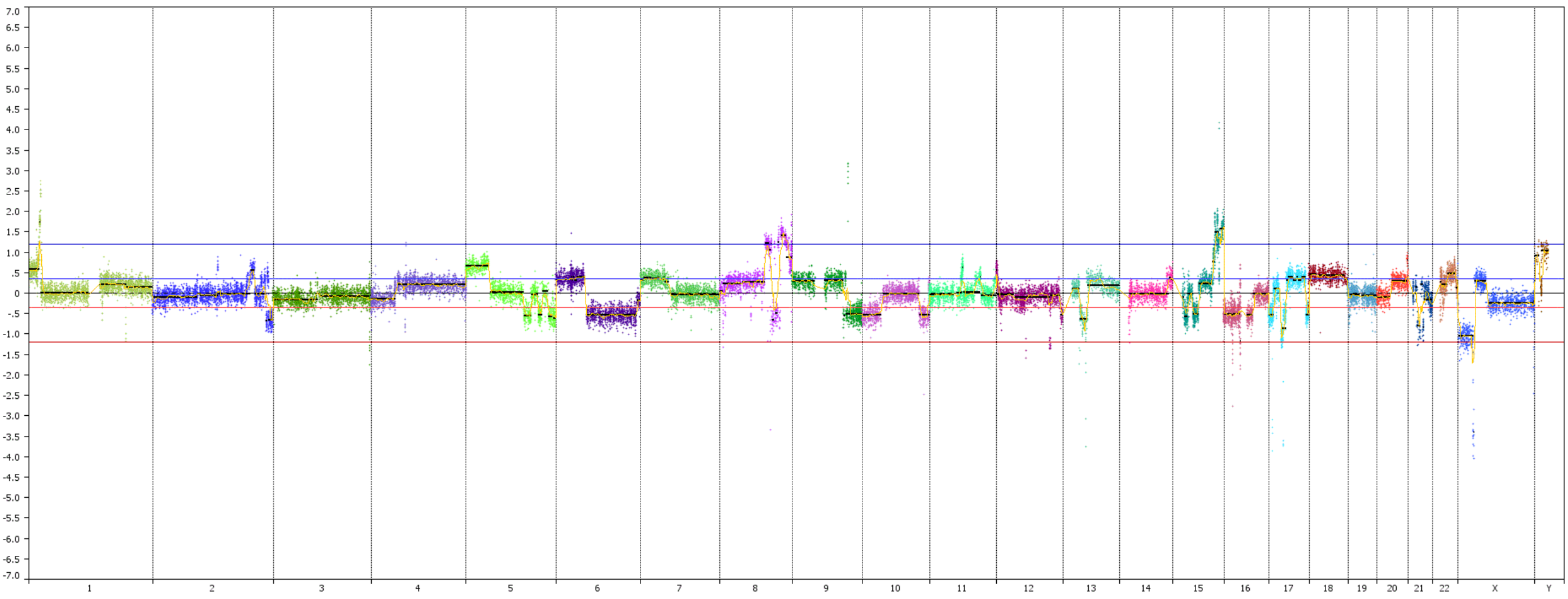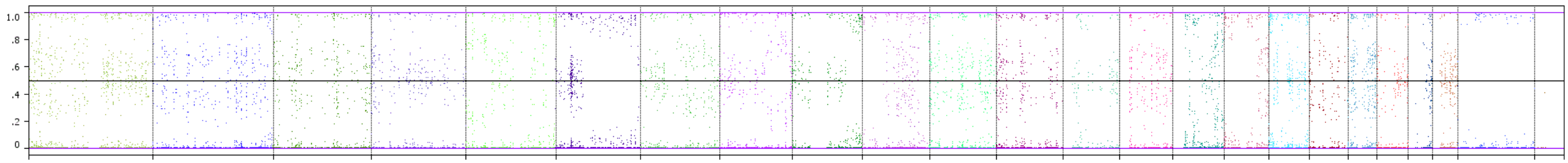

Supplement: Supplementary file 1 [file ijms-24-10463-s001.zip › Supplementary Document S1.pdf]
